# Multilevel proteomic analyses reveal molecular diversity between diffuse-type and intestinal-type gastric cancer

**DOI:** 10.1038/s41467-023-35797-6

**Published:** 2023-02-14

**Authors:** Wenhao Shi, Yushen Wang, Chen Xu, Yan Li, Sai Ge, Bin Bai, Kecheng Zhang, Yunzhi Wang, Nairen Zheng, Juan Wang, Shiqi Wang, Gang Ji, Jipeng Li, Yongzhan Nie, Wenquan Liang, Xiaosong Wu, Jianxin Cui, Yi Wang, Lin Chen, Qingchuan Zhao, Lin Shen, Fuchu He, Jun Qin, Chen Ding

**Affiliations:** 1 School of Life Sciences, Tsinghua University, Beijing, 100084 China; 2 State Key Laboratory of Proteomics, Beijing Proteome Research Center, National Center for Protein Sciences (The PHOENIX center, Beijing), Beijing Institute of Lifeomics, Beijing, 102206 China; 3 Department of Pathology, Zhongshan Hospital, Fudan University, Shanghai, 200032 China; 4 State Key Laboratory of Genetic Engineering and Collaborative Innovation Center for Genetics and Development, School of Life Sciences, Institute of Biomedical Sciences, Human Phenome Institute, Zhongshan Hospital, Fudan University, Shanghai, 200433 China; 5 Key Laboratory of Carcinogenesis and Translational Research (Ministry of Education), Peking University Cancer Hospital and Institute, Beijing, 100142 China; 6 State Key Laboratory of Cancer Biology, National Clinical Research Center for Digestive Diseases and Department of Digestive Surgery, Xijing Hospital of Digestive Diseases, Fourth Military Medical University, Xi’an, 710032 China; 7 Department of General Surgery & Institute of General Surgery, Chinese PLA General Hospital First Medical Center, Beijing, 100853 China; 8 Research Unit of Proteomics Driven Cancer Precision Medicine, Chinese Academy of Medical Sciences, Beijing, 102206 China

**Keywords:** Gastric cancer, Tumour heterogeneity, Cell signalling, Proteomics

## Abstract

Diffuse-type gastric cancer (DGC) and intestinal-type gastric cancer (IGC) are the major histological types of gastric cancer (GC). The molecular mechanism underlying DGC and IGC differences are poorly understood. In this research, we carry out multilevel proteomic analyses, including proteome, phospho-proteome, and transcription factor (TF) activity profiles, of 196 cases covering DGC and IGC in Chinese patients. Integrative proteogenomic analysis reveals *ARIDIA* mutation associated with opposite prognostic effects between DGC and IGC, via diverse influences on their corresponding proteomes. Systematical comparison and consensus clustering analysis identify three subtypes of DGC and IGC, respectively, based on distinct patterns of the cell cycle, extracellular matrix organization, and immune response-related proteins expression. TF activity-based subtypes demonstrate that the disease progressions of DGC and IGC were regulated by SWI/SNF and NFKB complexes. Furthermore, inferred immune cell infiltration and immune clustering show Th1/Th2 ratio is an indicator for immunotherapeutic effectiveness, which is validated in an independent GC anti-PD1 therapeutic patient group. Our multilevel proteomic analyses enable a more comprehensive understanding of GC and can further advance the precision medicine.

## Abstract (web summary)

The molecular differences between the two major gastric cancer subtypes diffuse-type gastric cancer (DGC) and intestinaltype gastric cancer (IGC) remain to be investigated. Here, integrated analysis of proteome, phospho-proteome and transcription factor activity for DGC and IGC reveals potential subtypes.

## Introduction

Gastric cancer (GC) is the third most common cause of global cancer mortality^1,2^. Gastric adenocarcinomas constitute ~95% of the GCs and are classified into diffuse, intestinal, and mixed types as per the widely used Lauren classification^3^. Patients diagnosed with diffuse-type GC (DGC) and those diagnosed with intestinal-type GC (IGC) account for 30% and 54% of all GC patients, respectively^4^. DGC displays a scattered cellular organization, poor adhesion, and poor cellular differentiation, while IGC displays a tubular or glandular cellular organization with tight adhesion junctions and less stromal component^3,4^. The different pathophysiological and molecular features of DGC and IGC suggest different mechanisms of carcinogenesis; therefore, it is imperative to investigate the mechanism differences between DGC and IGC.

In the past decade, large-scale genomic and transcriptomic studies carried out have revealed molecular characteristics of GC^5–7^. For example, the Cancer Genome Atlas (TCGA) conducted whole-exome sequencing and mRNA sequencing analyses of GC^5–7^. However, studies focused on systematical comparison of DGC and IGC were sparse. As reported in a study based on transcriptome analysis, Jinawath et al., found that genes encoding extracellular matrix (ECM) proteins were more highly expressed in DGC than in IGC, while those encoding metabolic proteins were more highly expressed in IGC than in DGC^8^. While there has been significant progress, deeper understanding of different molecular pathology of DGC and IGC from proteomic data remains lacking, which impedes the discovery of new biomarkers and drug targets for DGC and IGC.

Previous proteomic studies focused on the proteomic landscape of DGC^9,10^, which was a pathological type with poor prognosis and few treatment options^4^. For example, Ge et al. identified the proteomic subtypes and signaling pathways associated with clinical outcomes of DGC patients, such as cell cycle, epithelial-to-mesenchymal transition (EMT), and immune responses^9^. This demonstrates that DGC is characterized by inter-patient heterogeneity at protein level and can be classified based on proteomic signatures. Mun et al., used proteomic and phospho-proteomic approaches to systematically demonstrate alterations in key biological processes, such as cell proliferation, immune response, metabolism, and invasion of DGC^10^. These studies have significantly enhanced our understanding of the molecular heterogeneity prevalent in DGC. However, research investigating the underlying molecular subtypes of IGC is still lacking, despite IGC patients accounting for the highest proportion of total GC patients. Lack of clinical proteomic research on IGC hinders our comprehensive understanding of GC heterogeneity and searching for novel therapeutic targets.

The transcription factor (TF) activities orchestrate the intracellular signaling pathways during diverse biological processes in carcinogenesis. Several TFs have been reported to promote GC progression by different molecular mechanisms; for example, MYC promotes cell proliferation^11^; FOXC1 promotes EMT^12^; the SWI/SNF complex governs chromatin structure and gene transcription^13,14^; and the NFKB complex promotes inflammation and immune response^15^. However, the complete TF activity profiles of DGC and IGC have not been described yet. We have previously developed an approach called TFRE, which could detect and evaluate inferred TF activities at proteomic level^16,17^. Comprehensive analysis with TF activity profiles would provide a panoramic view of possible pathogenic mechanisms and therapeutics of DGC and IGC.

In addition to altered intracellular signal transduction induced by overexpression of TFs, components of the tumor microenvironment (TME) also affect disease progression in GC^18,19^. Recent studies have demonstrated that the TME is a complex system wherein the tumor-infiltrating immune cells play a key role in GC progression^18,19^. Moreover, heterogeneity of the TME affects immunotherapeutic effectiveness. Pembrolizumab, a monoclonal antibody directing against PD-1, is approved by US Food and Drug Administration (FDA) for advanced GC patients; however, the response rate is as low as 10–26% in GC patients with metastasis^20^. Therefore, the identification of predictive biomarkers and exploration of resistance mechanism to immunotherapy would be important for improving therapeutic effects for GC patients.

Here, we present multilevel proteomic analyses of GC by analyzing the proteome of 196 pairs of tumor tissues and their normal adjacent tissues (NATs). We demonstrate the different pathogenic mechanisms between DGC and IGC based on multi-omics data. Moreover, we perform proteomic clustering and obtain molecular subtypes with distinct expression levels of proteins that play a role in cell cycle, ECM, and immune response, indicating the heterogeneity prevalent in DGC and IGC. Additionally, we find that NFKB and SWI/SNF complexes are crucial in distinguishing two subtypes of DGC and IGC, and are associated with different patient prognosis, respectively. The characterization of immune landscape further reveals the existence of diverse immunotherapy targets, especially for Th1/Th2 ratio in predicting GC immunotherapeutic effectiveness. Our integrative proteomic analyses present a multilevel proteomic landscape that serve as a rich resource for understanding the molecular characteristics of GC and for identifying potential therapeutic targets in GC treatment.

## Results

### Comprehensive proteomic landscape of GC cohort

**Fig. 1:**
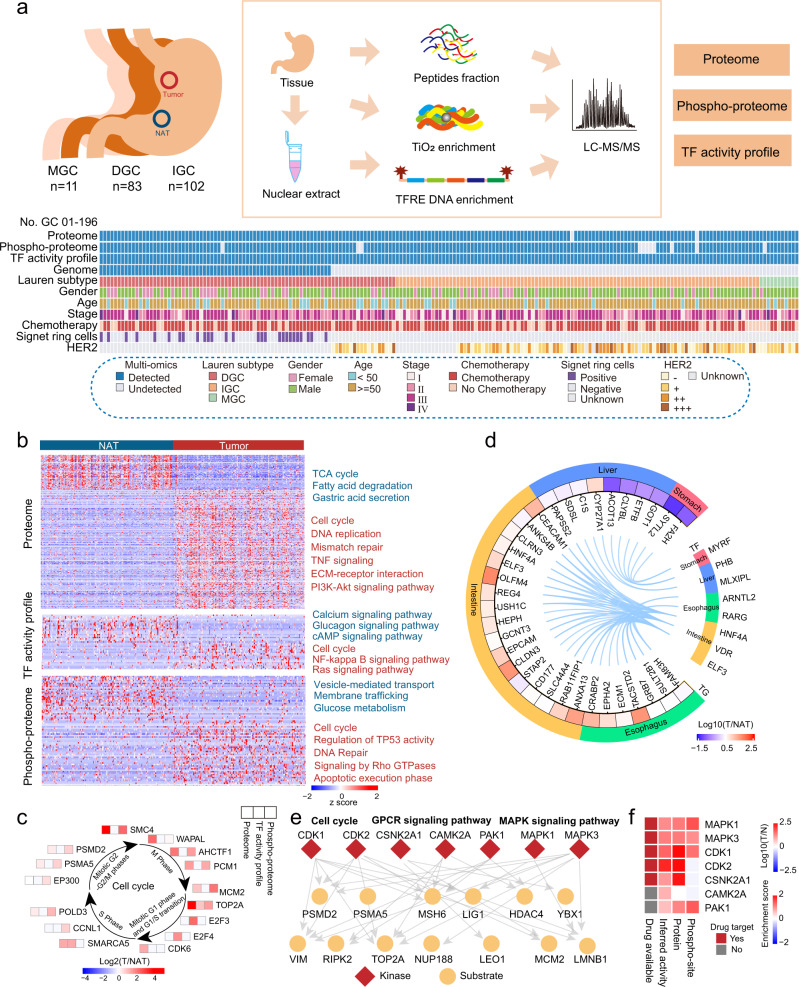
Multilevel proteomic atlas of human GC samples. **a** Workflow of human gastric cancer multilevel proteomic atlas construction. **b** Differentially expressed proteins in tumor tissues and NATs and their associated biological pathways. Red, upregulated pathways in tumor tissues; blue, upregulated pathways in NATs. **c** Representative differentially expressed proteins in the cell cycle with multilevel proteomic levels (proteome, TF activity profile, and phospho-proteome). **d** Regulation network of TFs and their target genes. Tissue-specific TFs are shown. **e** Phospho-regulatory network in GC. Red, kinases; yellow, substrates. The main function or pathways of substrate proteins are labeled. **f** The expression of GC signature kinases in multilevel proteomic level (protein, phospho-site, and inferred activity).

We collected 196 pairs of primary GC samples (DGC, *n* = 83; IGC, *n* = 102; and mixed-type gastric cancer (MGC), *n* = 11) and the NATs from treatment-naïve Chinese patients (Supplementary Table 1, Supplementary Data 1). A schematic of the experimental design is shown in Fig. 1a. A mass spectrometry (MS)-based label-free quantification strategy, referred to the Chinese Human Proteome Project (CNHPP)^21–23^, was adopted for this study. A Fast-Seq workflow^24^ was performed to profile the proteomes of 194 paired samples. A phospho-proteomic analysis was conducted on 184 paired samples using a TiO_2_ enrichment strategy^25^. In addition, concatenated tandem array of consensus TF response elements (TFRE) for TF enrichment, reflecting TFs’ DNA binding activity, was carried out for all the samples^16^. The tryptic digestions of the 293T cell lysate were measured as standards to evaluate sample quality control (QC). The average spearman’s correlation coefficients among standards in proteome, phospho-proteome, and TF activity profile platforms were 0.92, 0.94, and 0.95 (Supplementary Fig. 1a), respectively. The median coefficient of variation (CV) values among standards in proteome, phospho-proteome, and TF activity profile platforms were 0.28, 0.26, and 0.34 (Supplementary Fig. 1b), respectively. The density of three datasets exhibited unimodal distribution (Supplementary Fig. 1c). These evaluations demonstrated the stability of our MS platforms.

Upon profiling the proteomes of all the patient samples, we identified 11,688 proteins in total (Supplementary Fig. 1d). Furthermore, 44,750 phospho-sites were identified for 6619 phosphoproteins with a confident site localization score (Mascot ion score >20, Supplementary Fig. 1d), and 597 TFs were identified upon the inferred TF activity profiles (Supplementary Fig. 1d). The pairwise samples review showed that the number of proteins, phospho-sites, and TFs identified in the GC tumor samples were higher than that identified in the paired NAT samples (Supplementary Fig. 1d, e). This suggested a lower degree of differentiation and a higher degree of heterogeneity in tumor tissues than that in the NATs. Multilevel proteomics increased the proteome coverage of kinases and TFs. For example, phospho-proteomics increased the number of kinases detected to 325, and the TF activity profiles increased the number of TFs detected to 756 (Supplementary Fig. 1f).

Our multilevel proteomic data enabled a comprehensive exploration of altered protein expression between the GC tissues and NATs. After sample QC and normalization procedures, we performed principal-component analyses (PCAs) of proteomes, phospho-proteomes and TF activity profiles. All datasets could separate tumors and NATs (Supplementary Fig. 2a), indicating altered proteins, phospho-sites, and TF activity landscape in GC. Compared with the NATs, GC-related proteins, TFs, and phospho-sites were identified. Among them, 1548 proteins, 123 TFs, and 163 phospho-sites were upregulated, and 671 proteins, 20 TFs, and 194 phospho-sites were downregulated (two-sided Wilcoxon signed-rank test, BH adjusted *p* < 0.05, ratio of tumor to NAT (T/NAT) > 2 or <0.5, Fig. 1b, Supplementary Data 3a–c) in tumor tissues. Gene set enrichment analysis (GSEA) of proteome demonstrated that the proteins involved in DNA replication, cell cycle, ECM organization, and immune response were significantly upregulated in tumor tissues, whereas those involved in metabolism (i.e., fatty acid β-oxidation, tricarboxylic acid (TCA) cycle, and oxidative phosphorylation) were significantly downregulated in tumor tissues (Fig. 1b, Supplementary Fig. 2b). Pathway enrichment analysis of TF activity profiles indicated that upregulated TFs in tumor tissues involved in mediating cell cycle, NF-kappa B signaling pathway, and Ras signaling pathway, whereas upregulated TFs in NATs involved in calcium signaling pathway, glucagon signaling pathway, and cAMP signaling pathway (Fig. 1b). Pathway enrichment analysis of phospho-proteome indicated that tumor phosphoproteins were involved in carcinogenesis-associated pathways/processes, such as the cell cycle, regulation of TP53 activity, and DNA repair, et al., whereas those in NATs were involved in physiological functions, such as vesicle-mediated transport, membrane trafficking, and glucose metabolism (Fig. 1b). These analyses showed that the characteristics of tumor tissues at proteome, TF activity, and phospho-proteome levels showed partial consistency with some differences.

Notably, proteins involved in the cell cycle were upregulated in all the three datasets. Cell cycle proteins were evaluated based on their altered expression patterns (WAPAL, AHCTF1, etc.), phosphorylation patterns (CCNL1 T67, SMC4 S41, etc.), and inferred TF activities (SMARCA5, E2F3, etc.) in GC tumor tissues (Fig. 1c). We found that GC patients with high DNA binding activities of SMARCA5 and E2F3 and high phosphorylation of CCNL1 (at T67) and SMC4 (at S41) in tumor tissues associated with poor prognoses (Log-rank test, *p* < 0.05) (Supplementary Fig. 2c). The analyses in TF activity profiles and phospho-proteomes suggested that TFs and kinases played a unique role in oncogenesis. This is a finding that cannot be observed from merely analyzing the protein expression profiles.

A recent analysis on the GC cell lineage revealed that GC cells may transdifferentiate into other digestive tract cell lineages^26^. As TFs determined the cell fate^27^, firstly, we compared inferred TF activities between the tumor tissues and NATs and found that intestine-specific TFs, including ELF3, HNF4A, and VDR, and esophagus-specific TFs, including RARG and ARNTL2, were upregulated in tumor tissues; however, stomach-specific TFs, such as MYRF, were downregulated (Supplementary Fig. 2d). Secondly, we compared these TFs’ target genes (TGs) expression between the tumor tissues and NATs based on proteomic profile. As presented in Fig. 1d, the TF-TG regulation network showed that TGs expression levels exhibited similar tendencies with tissue-specific TFs. Thirdly, we comprehensively counted the proportion of tissue-specific-proteins expression changes between GC tumor- and NAT-specific protein expression. We found that 25.0% intestine- and 37.8% esophagus-specific proteins, such as ELF3 and EPHA2, respectively, were upregulated in the tumor tissues, whereas 77.1% stomach-specific proteins, such as MUC5AC, were downregulated (Supplementary Fig. 2e, Supplementary Data 3d). The downregulated stomach-specific proteins in GC tumor tissues (Supplementary Fig. 2f) indicated that the normal physiological function of stomach decreased or lost in GC tumor tissues. At last, we analyzed the association between stomach-specific proteins expression and GC patients’ prognosis. We found stomach-specific proteins, such as VSIG2 and B4GALNT3, were associated with favorable prognosis (Log-rank test, *p* < 0.05; Supplementary Fig. 2g). These results demonstrated that the altered expression of tissue-specific TFs affected trans-differentiation in GC and patients’ prognosis, reinforcing the fact that proteomic, phospho-proteomic, and TF activity profiles possessed distinct biological characteristics. Comprehensive analysis of multilevel proteomics could provide novel insights into signaling pathways and drug targets.

To explore the role of kinases in GC, we selected phospho-sites which exhibited larger alteration than its protein expression alteration between the tumor tissues and NATs. Subsequently, 229 phospho-sites were defined as GC-associated phospho-sites (Supplementary Fig. 2h). Kinase substrate enrichment analysis (KSEA)^28^ of GC-associated phospho-sites identified multiple kinases, including CDK1, CDK2, CSNK2A1, CAMK2A, PAK1, MAPK1, and MAPK3, were activated in GC tumor tissues (Fig. 1e). These kinases regulated cell cycle and several oncogenic pathways, including GPCR signaling pathway and MAPK signaling pathway. Further investigation revealed that the expression, phosphorylation, and activity of MAPK1, MAPK3, and CDK1 had increased; thus, these three kinases could serve as potential drug targets for GC patients (Fig. 1f).

Thus, our findings have so far established a comprehensive proteomic landscape of Chinese GC patients. Moreover, these datasets serve as a multilevel resource for investigating GC pathology and precision medicine.

### *ARID1A* mutation performed different effects between DGC and IGC

**Fig. 2:**
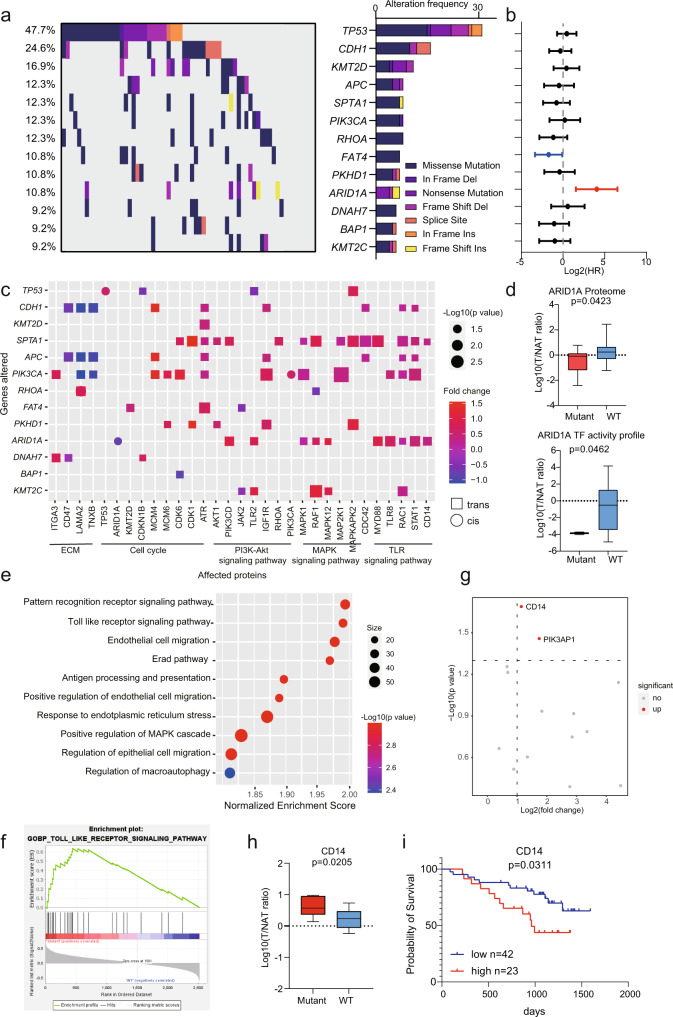
A summary of proteogenomic analysis of DGC. **a** Genes with non-silent variants in at least six patients (9%) are depicted on the OncoPrint. Bars on the right of the graph show the numbers of non-synonymous mutations. **b** Prognosis outcomes of corresponding gene mutations. *n* (with *ARID1A* mutant) = 7 and *n* (without *ARID1A* mutant) = 58 biologically independent samples. The points and error bars show the median of hazard ratio (HR) and 95% confidence interval (CI). **c** *Cis-* and *trans*-*effects* of significantly mutated genes (*y*-axis) on protein level (*x*-axis). The *p*-values are calculated by Fisher’s exact test. The related biological functions and pathways are shown at the bottom. **d** Altered expression of ARID1A associated with *ARID1A* mutations. In proteome, *n* (mutant) = 7 and *n* (WT) = 58 biologically independent samples. In TF activity profile, *n* (mutant) = 2 and *n* (WT) = 38 biologically independent samples. Boxplots show median (central line), upper and lower quartiles (box limits), min to max range. The *p*-values are calculated using two-sided student’s *t*-test. **e** Pathways enriched by GSEA in *ARID1A* mutated patients. Nominal *p*-value, calculated as phenotype based permutation test. **f** TLR signaling pathway was significantly enriched in *ARID1A* mutated patients. **g** Altered expression of proteins enriched in TLR signaling pathway. The *p*-values are calculated by two-sided Wilcoxon rank-sum test. **h** Altered expression of CD14 associated with *ARID1A* mutation. *n* (mutant) = 7 and *n* (WT) = 58 biologically independent samples. Boxplots show median (central line), upper and lower quartiles (box limits), min to max range. The *p*-value is calculated using two-sided student’s *t*-test. **i** The prognostic outcome of CD14 in DGC. *n* (low) = 42 and *n* (high) = 23 biologically independent samples. The *p*-value is calculated using Log-rank test. Source data are provided as a Source Data file.

To investigate the alteration of genetic information of GC, we performed statistical analysis on gene mutation frequency based on the panel of 274 cancer driver and GC hotspot genes among 65 DGC patients. Thirteen genes detected with mutations in at least 9% patients were presented (Fig. 2a, Supplementary Data 2). Among these gene mutations, *TP53, CDH1, KMT2D, RHOA, ARID1A, APC*, and *PIK3CA* were detected as high-frequency mutations (10.8-47.7%), consistent with previous reports^5,9^. To explore the association of gene mutations with prognostic outcomes, we calculated the hazard ratio (HR) of gene mutations based on survival outcomes. We found that patients with *ARID1A* mutation had unfavorable prognosis (Fig. 2b).

Genomic alterations that affect gene expression levels at the same locus are designated as *cis-effects*, whereas an impact of another locus is defined as a *trans-effect*^29^. We comprehensively characterized the *cis*- and *trans-effects* of genetic alterations on protein level (Fig. 2c). Comparing to *cis-effects*, protein abundance alterations occurred more prominently in numerous *trans-effects*, and these alterations had biological process propensity. Consistently, patients with *CDH1* mutation had lower ECM proteins expression (Fig. 2c), which showed the *CDH1* gene function in ECM organization^30^. Importantly, only three genes showed *cis-effects*: *TP53*, *PIK3CA*, and *ARID1A*. Patients with the *TP53* or *PIK3CA* mutations had increased corresponding proteins abundance, while patients with *ARID1A* mutation had lower ARID1A protein expression (Fig. 2d). We also compared the TF activity of ARID1A in TF activity profiles between 7 patients with *ARID1A* mutation and wild type patients. We found that the TF activity of ARID1A was also decreased in *ARID1A* mutated patients (Fig. 2d). These results demonstrated that *ARID1A* mutation caused its protein expression decrease and TF activity reduction.

As the only gene mutation which was correlated with unfavorable prognosis, we further investigated how *ARID1A* mutation correlated with the alteration of the cancer proteome, namely alterations of related proteins and pathways. We mined TGs’ data of ARID1A^31^. ARID1A was primarily reported as a transcriptional repressor^32^. Thus, we surveyed the TGs that were elevated in *ARID1A* mutated patients. GSEA analysis showed significantly altered pathways between samples with and without *ARID1A* mutation. Based on the normalized enrichment scores, we found that pattern recognition receptor signaling pathway and TLR signaling pathway were the most significantly enriched pathways in *ARID1A* mutated patients (Fig. 2e–f). Among 15 proteins involved in TLR signaling pathway, CD14 and PIK3AP1 were significantly upregulated in *ARID1A* mutated patients (Fig. 2g, h). Furthermore, the prognostic analysis showed that CD14 was an unfavorable prognostic protein in DGC (Log-rank test, *p* < 0.05; Fig. 2i). CD14 had been reported as a protein involved in increasing cytokine production, increasing tumor growth, and promoting inflammatory in several cancer types^33^. These results demonstrated that patients with *ARID1A* mutation had unfavorable outcomes and activated CD14 mediated TLR signaling pathway.

The *ARID1A* mutation was found as an unfavorable prognostic factor in this DGC cohort. Then, we surveyed the prognostic correlation of *ARID1A* mutation in IGC cohort. In Wang’s cohort, we found *ARID1A* mutation was a prognostic factor associated with better prognosis^34^. Then, we explored the TCGA cohort^7^, validating prognostic association of *ARID1A* mutation, and found patients with *ARID1A* mutation in IGC had better prognoses, whereas patients with *ARID1A* mutation in DGC were associated with poor prognoses (Supplementary Fig. 3). These results indicated that the mutation of *ARID1A* had opposite prognostic effects between DGC and IGC, via diverse influences on their corresponding proteomes. Therefore, it is important to compare DGC and IGC based on multilevel proteomic data.

### Integrated multilevel proteomics in DGC and IGC

**Fig. 3:**
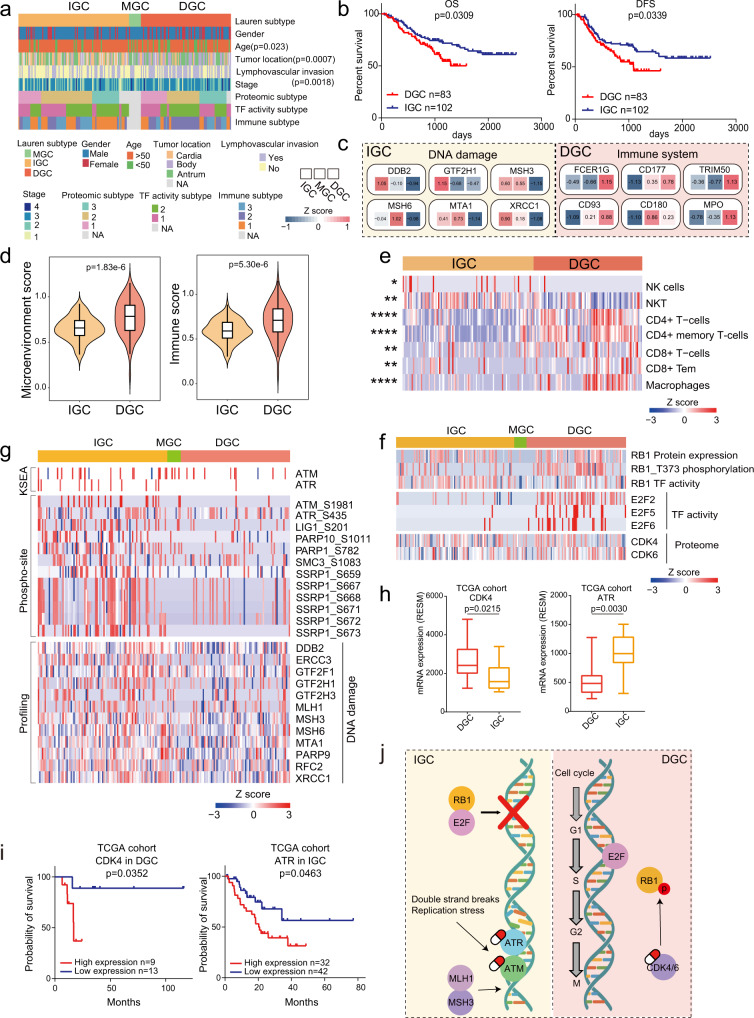
Integrated multilevel proteomic analyses showed different pathogenic mechanism of DGC and IGC. **a** The association of Lauren classification with clinical information. Two-sided Fisher’s exact test is used for categorical variables. **b** The association of Lauren classification with clinical outcomes. *n* (DGC) = 83 and *n* (IGC) = 102 biologically independent samples. *P*-values are from Log-rank test. **c** Representative differentially expressed proteins in the featured pathways of DGC and IGC. **d** Microenvironment scores and immune scores of DGC and IGC. *n* (DGC) = 83 and *n* (IGC) = 100 biologically independent samples. Violin plots show median and interquartile range. The *p*-values are from two-sided Wilcoxon rank-sum test. **e** Comparison of immune cell infiltration between DGC and IGC. Two-sided Wilcoxon rank-sum test is used. The Benjamini–Hochberg (BH) adjusted *p*-values are 0.019 (NK cells), 0.0016 (NKT), 1.71E-9 (CD4 + T-cells), 1.89E-12 (CD4 + memory T-cells), 0.0017 (CD8 + T-cells), 0.00056 (CD8 + Tem), and 4.25E-7 (Macrophages). **f** Integrated analysis of cell cycle regulation pathway at protein, kinase, TF activity and phospho-site levels in DGC and IGC. **g** Integrated analysis of DNA mismatch repair pathway at protein, kinase and phospho-site levels in DGC and IGC. **h** The expression of CDK4 and ATR in DGC and IGC. *n* (DGC) = 10 and *n* (IGC) = 16 biologically independent samples. Boxplots show median (central line), upper and lower quartiles (box limits), min to max range. The *p*-values are calculated by two-sided Wilcoxon rank-sum test. **i** The prognostic analyses of CDK4 and ATR in TCGA cohort. In comparison of CDK4, *n* (high expression) = 9 and *n* (low expression) = 13 biologically independent samples. In comparison of ATR, *n* (high expression) = 32 and *n* (low expression) = 42 biologically independent samples. The *p*-value is calculated using Log-rank test. **j** Summary of signature proteins and pathways involved in DGC and IGC. ****p < 1.0e-4, ***p < 1.0e-3, **p < 0.01, *p < 0.05. Source data are provided as a Source Data file.

Lauren classification includes DGC, IGC, and MGC, among which the former two pathological types are the major^3^. In our cohort, clinical information showed that DGC development was significantly dependent on age (Chi-square test, BH adjusted *p* = 0.023), tumor location (Chi-square test, BH adjusted *p* = 0.0007), and lymphovascular invasion (Chi-square test, BH adjusted *p* = 0.0018; Fig. 3a). Consistent with the current clinical knowledge, survival analysis revealed that IGC patients had significantly prolonged survival (Log-rank test, *p* = 0.0309; Fig. 3b).

To investigate altered proteomic features of GC, we compared proteins with significantly differential expression (Wilcoxon paired signed-rank test, BH adjusted *p* < 0.05, foldchange > 1.5) between tumor tissues and NATs in DGC and IGC, respectively (Supplementary Fig. 4a). We identified 2512 proteins upregulated in DGC, among which 2212 (88.1%) proteins were also upregulated in IGC (68.2% of upregulated proteins in IGC). Among 1106 proteins downregulated in DGC, 686 (62.0%) proteins were also downregulated in IGC (82.2% of downregulated proteins in IGC). Based on T/NAT ratio, we further filtered out 1897 differentially expressed proteins between DGC and IGC (2 folds), and divided them into six groups (Supplementary Fig. 4b). Proteins in group 1 (449 proteins) and group 2 (921 proteins) were upregulated in tumor tissues in DGC and IGC. Proteins in group 4 (374 proteins) and group 5 (142 proteins) were downregulated in tumor tissues in DGC and IGC. Most differential proteins (1886/1898) were included in these four groups, and the pathways enriched in these four groups were shown in Supplementary Fig. 4c. Proteins in group 3 (10 proteins) and proteins in group 6 (1 protein) had converse dysregulation directions between IGC and DGC (Supplementary Fig. 4d). The prognostic analysis (Supplementary Fig. 4e) revealed that GALNT3 and TRMT10C were oppositely associated with prognostic outcomes in DGC and IGC, which deserved to be further studied. These results demonstrated that, when comparing to NATs, the directions of most dysregulations in DGC and IGC tumor tissues were consistent, while the change magnitudes of the differential proteins were different between DGC and IGC. Overall, DGC and IGC tumor tissues showed tumorous characteristics compared to NATs, while the comparison based on T/NAT ratio showed tumor heterogeneity between DGC and IGC.

To further investigate the tumor heterogeneity between DGC and IGC, we compared the differential expression at multilevel (proteome, phospho-proteome, and TF activity). Based on the ratio of tumor tissues to NATs, we found a total of 384 proteins differentially expressed between DGC and IGC (two-sided Wilcoxon rank-sum test, BH adjusted *p* < 0.05, ratio of DGC to IGC > 2 or <0.5). Among them, 83 and 301 were upregulated in DGC and IGC, respectively (Fig. 3c, Supplementary Fig. 5a, b, Supplementary Data 4a). Pathway enrichment analysis demonstrated that the significantly upregulated proteins in DGC were involved in immune system, complement cascade, ECM organization, and cell migration, suggesting that the TME proteins were major components of the DGC proteome. In contrast, proteins upregulated in IGC were mainly involved in DNA damage, ERBB signaling, metabolism, and VEGF signaling pathway. Additionally, 743 and 536 phospho-sites were enriched in DGC and IGC, respectively (two-sided Wilcoxon rank-sum test, BH adjusted *p* < 0.05, ratio of DGC to IGC > 2 or <0.5; Supplementary Data 4b). The pathway enrichment of phospho-proteome validated that ECM organization, immune system, and metastasis played major roles in DGC progression, while proliferation and metabolism played major roles in IGC progression (Supplementary Fig. 5c).

To explore the different effects of tumor microenvironment in DGC, we compared the xCell scores^35^ of the DGC and IGC tumors. The microenvironment and immune scores were higher in DGC than in IGC (Wilcoxon rank-sum test, BH adjusted *p* < 0.05; Fig. 3d), indicating a higher degree of tumor infiltration by immune cells in DGC than in IGC. Subsequently, we compared immune cells prevalent in DGC and IGC tumors and found that DGC tumors had higher infiltration of CD4 + T cells, CD8 + T cells, and macrophages than the IGC tumors (Wilcoxon rank-sum test, BH adjusted *p* < 0.05, Fig. 3e).

Pathway enrichment analysis showed cell cycle related processes were upregulated in both DGC and IGC, but the specific signaling pathways were different (Supplementary Fig. 5c). We then investigated the different molecular mechanisms related to cell cycle in DGC and IGC, to search for the distinct potential drug targets. RB1 is a crucial TF that suppresses cell cycle by inhibiting E2F in tumors. Phosphorylation of RB1 by CDK4/6 causes the dissociation of E2F from the RB1-E2F complex, releasing RB1-regulated cell cycle suppression^36^. For DGC patients, we observed that RB1 possessed increased phosphorylation and decreased TF activity compared with IGC patients, while E2F activity and CDK4/6 levels were upregulated in DGC (Fig. 3f). This integrative analysis demonstrated that RB1 was phosphorylated in DGC and promoted the disassociated with E2F, which increased E2F activity and drove cell cycle progression in DGC. These results indicated the possibility of employing the CDK4/6 complex as a potential drug target for DGC.

For IGC patients, we performed a comprehensive investigation of the DNA repair network by evaluating the kinase activity, phospho-site, and protein expression levels. Twelve proteins involved in DNA damage, including MLH1, MSH3, and MSH6, were upregulated in IGC patients (Fig. 3g, Wilcoxon rank-sum test, *p* < 0.05, ratio of IGC to DGC > 2). Further investigation showed increase in phospho-sites on DNA damage proteins in IGC patients, including those on PARP1, SMC3, and SSRP1. Additionally, our comparative analysis indicated that phosphorylated ATM/ATR were upregulated in IGC patients (Fig. 3g). Previous studies have indicated that ATM/ATR, core components of the DNA repair network, are activated to initiate homologous recombination repair in the event of DNA double-strand breaks^37^. Thus, we can presume that DNA damage related proteins can serve as potential drug targets for IGC. In order to further validate these findings, we validated these potential drug targets of DGC and IGC in TCGA cohort^7^. We compared the expression of CDK4/6 and ATM/ATR, and found the expression of CDK4 was higher in DGC, and the expression of ATR was higher in IGC (Fig. 3h). Further, prognostic analysis showed the expression of CDK4 and ATR were both negatively associated with clinical outcomes in DGC and IGC (Fig. 3i), respectively. These results proved that CDK4/6 and ATM/ATR were the potential targets for DGC and IGC, respectively (Fig. 3j).

PCAs of TF activity profiles could distinguish between the DGC and IGC datasets (Supplementary Fig. 5d), indicating a large difference in molecular features between DGC and IGC. We reasoned that certain key TFs would be not only upregulated in tumor tissues in comparison to NATs, but elevated in particular tumor subtypes. We compared TF activities between DGC and IGC and found that 24 TFs were differentially activated between DGC and IGC (two-sided Wilcoxon rank-sum test, BH adjusted *p* < 0.05, ratio of DGC to IGC > 2 or <0.5; Supplementary Fig. 5e). Among them, 20 and 4 TFs were upregulated in DGC and IGC, respectively. Subsequently, we proposed the concept master TFs, which could be further predicted by the enrichment of corresponding downstream TGs based on CellNet database^38^. As a result, FOXC1 and MYC were regarded as the master TFs of DGC and IGC, respectively (Supplementary Fig. 5f, g, Supplementary Data 4c). Further analysis of TGs regulated by master TFs suggested that TGs of FOXC1 were mainly involved in ECM organization (COL1A1 and COL1A2), ECM-receptor interaction (COL4A1/2, and LAMB2), and migration (WNT5A), which were dominated in DGC; while TGs of MYC were mainly involved in ribosome biogenesis (IMP4 and NOP56), RNA metabolism (PUS1 and RPL13A), and proliferation (FBL and DKC1), which were dominated in IGC (Supplementary Fig. 5h, i). TG expression levels of LAMB2 and FBL were associated with poor clinical outcomes in DGC and IGC patients, respectively (Log-rank test, *p* < 0.05; Supplementary Fig. 5j). The TF activity analyses results were consistent with the finding that altered proteins were involved in proliferation and DNA damage in IGC, while ECM and immune response in DGC.

In summary, our comprehensive analysis based on multilevel proteomics provided profound mechanisms and proposed CDK4/6 and ATM/ATR as the potential targets for DGC and IGC, respectively.

### Proteomic subtypes of GC and their association with clinical outcomes

**Fig. 4:**
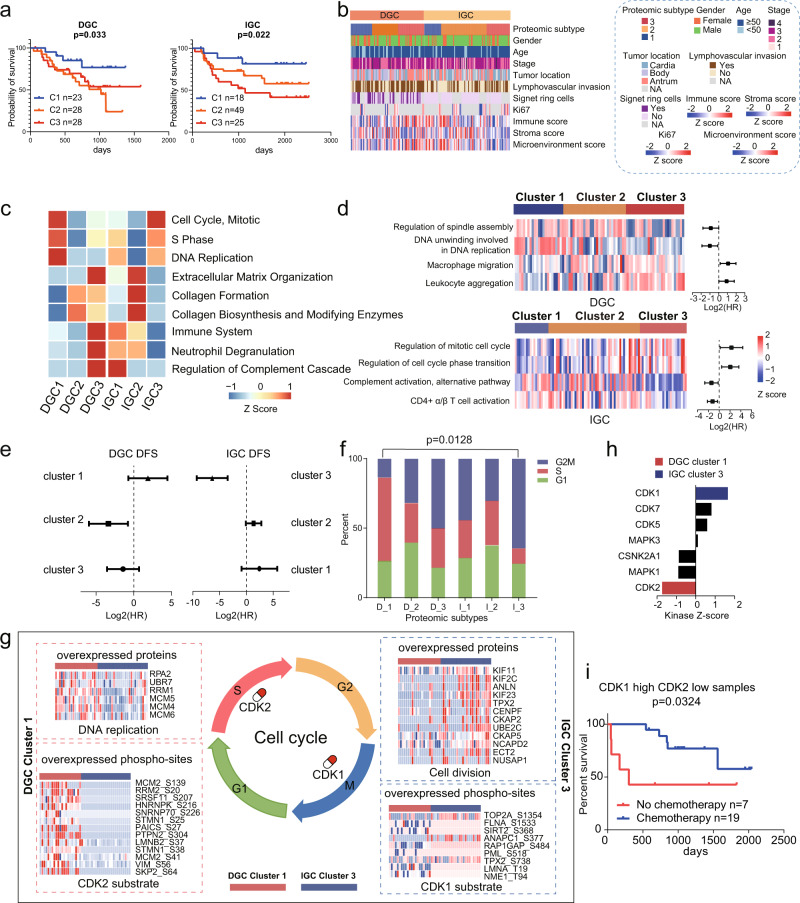
Proteomic subtyping of GC and associations with clinical outcomes. **a** The association of proteomic subtypes with clinical outcomes in DGC and IGC. *n* (DGC cluster 1) = 23, *n* (DGC cluster 2) = 28, *n* (DGC cluster 3) = 28, *n* (IGC cluster 1) = 18, *n* (IGC cluster 2) = 49, and *n* (IGC cluster 3) = 25 biologically independent samples. *P*-values are from Log-rank test. **b** Clinical characteristics annotation in GC proteomic subtypes. **c** Pathways that significantly enriched in the proteomic subtypes. **d** GSEA revealed the cell cycle and immune related pathways are enriched in the proteomic subtypes and have opposite prognoses between DGC and IGC. *n* (DGC) = 79 and *n* (IGC) = 92 biologically independent samples. The points and error bars show the median of hazard ratio (HR) and 95% confidence interval (CI). **e** The association of chemotherapy with DFS in each GC proteomic subtypes. Chemotherapy: *n* (DGC cluster 1) = 19, *n* (DGC cluster 2) = 22, *n* (DGC cluster 3) = 22, *n* (IGC cluster 1) = 12, *n* (IGC cluster 2) = 36, and *n* (IGC cluster 3) = 20 biologically independent samples. No chemotherapy: *n* (DGC cluster 1) = 4, *n* (DGC cluster 2) = 6, *n* (DGC cluster 3) = 7, *n* (IGC cluster 1) = 6, *n* (IGC cluster 2) = 13, and *n* (IGC cluster 3) = 5 biologically independent samples. The points and error bars show the median of hazard ratio (HR) and 95% confidence interval (CI). **f** Percentage of patients with different cell cycle phases. The *p*-value is calculated by Fisher’s exact test. **g** Summary of cell cycle regulation in DGC cluster 1 and IGC cluster 3. Proteins involved in DNA replication and cell division in DGC cluster 1 and IGC cluster 3, phospho-sites of CDK1 and CDK2 substrates in DGC cluster 1 and IGC cluster 3 are shown, respectively. **h** KSEA analysis of CDKs kinase activities in DGC cluster 1 and IGC cluster 2. Kinases with *p*-value < 0.05 (permutation test) are colored in red (CDK2, *p*-value = 0.041) or blue (CDK1, *p*-value = 0.049). **i** The assoc**i**ation of chemotherapy with DFS in GC patients with high CDK1 and low CDK2 level. *n* (chemotherapy) = 19 and *n* (no chemotherapy) = 7 biologically independent samples. The *p*-value is from Log-rank test. Source data are provided as a Source Data file.

Clinically, tumor treatment is largely depended on histological examination. Increasing numbers of studies have showed there were different molecular subtypes, which had different prognosis and therapeutic targets, in each tumor histological type^39,40^. Consensus clustering^41^ based on upregulated proteins in DGC tumor tissues compared with NATs identified three DGC proteomic subtypes: DGC cluster 1 (*n* = 23), DGC cluster 2 (*n* = 28), and DGC cluster 3 (*n* = 28). Similarly, we applied upregulated proteins in IGC tumors tissues compared with NATs in consensus clustering and identified three IGC proteomic subtypes: IGC cluster 1 (*n* = 18), IGC cluster 2 (*n* = 49), and IGC cluster 3 (*n* = 25) (Supplementary Fig. 6a). Multivariate cox regression analysis suggested that these subtypes were significantly associated with clinical outcomes, irrespective of other clinical characteristics, including gender, age, TNM stage, and chemotherapy (Log-rank test, *p* < 0.05; Fig. 4a, b). This result indicated that proteomic subtyping could serve as an independent prognostic predictive factor. Notably, for DGC patients, DGC cluster 1 had the best prognosis, whereas DGC cluster 2 and cluster 3 had worse prognoses; for IGC patients, IGC cluster 1 had the best prognoses, whereas IGC cluster 3 had the worst prognosis.

These subtypes showed distinct molecular features. We identified 2367 and 3154 differentially expressed proteins (two-sided Wilcoxon rank-sum test, BH adjusted *p* < 0.05, foldchange > 2) across the DGC and IGC clusters, respectively. Among the six subtypes, DGC cluster 1 and IGC cluster 3 were characterized by cell cycle (such as CDK1/2 and CDK6) and DNA replication (such as ORCS3 and AHCTF1); DGC cluster 2 and IGC cluster 2 were featured with ECM organization (such as DMD and MUC5AC), collagen formation and biosynthesis (such as CD36, COL6A1, and LAMA2); many immune response-related proteins (such as CD163, IDO1, and ICAM1), and proteins regulating neutrophil degranulation and complement cascade (such as FCER1G, IL 16, and C5) were overrepresented in DGC cluster 3 and IGC cluster 1 (Fig. 4c, Supplementary Fig. 6b, Supplementary Data 5a, b). Consistently, the DGC cluster 3 and IGC cluster 1 had the highest immune score in DGC and IGC, respectively; IGC cluster 2 had the highest stroma score in IGC (Fig. 4b). KSEA analysis within each cluster based on tumor phospho-proteomes revealed activation of subtype-specific kinases (Supplementary Fig. 7a, b, Supplementary Data 5c). We found PRKACA and PRKCA were activated in DGC cluster 3 and IGC cluster 1, respectively; TGFBR2 was activated both in IGC cluster 2 and DGC cluster 2; AURKB was activated both in IGC cluster 3 and DGC cluster 1. These observations suggested that DGC and IGC clusters exhibited distinct characteristics, which were validated in tumor microenvironment, pathway enrichment, and kinase enrichment. Among these subtypes, DGC cluster 3 and IGC cluster 1 had similar biological processes, while DGC cluster 1 and IGC cluster 3 showed more consistence.

We observed a contrasting phenomenon in this study wherein DGC and IGC patients with upregulated cell cycle- and immune response-related proteins exhibited different clinical outcomes. DGC cluster 1 featured with cell cycle had better prognosis than DGC cluster 3 featured with immune-response related processes. Conversely, IGC cluster 3, with similar molecular features as DGC cluster 1, had poor prognosis (Fig. 4a). ssGSEA analysis in these clusters further demonstrated that the NESs (normalized enriched scores) for cell cycle- and immune response-related signaling pathways were associated with different clinical outcomes in DGC and IGC (Fig. 4d). Regulation of spindle assembly and DNA unwinding involved in DNA replication were associated with favorable prognosis in DGC. However, the regulation of mitotic cell cycle and regulation of cell cycle phase transition were associated with unfavorable prognosis in IGC (Log-rank test, *p* < 0.05). Moreover, alternative complement pathway activation and CD4 + α/β T cell activation were associated with favorable prognosis in IGC, but macrophage migration and leukocyte aggregation were associated with unfavorable prognosis in DGC (Log-rank test, *p* < 0.05). Further, we validated this finding using the transcriptomic dataset of TCGA GC cohort^7^. We performed ssGSEA and calculated NES of pathways for every patient, and analyzed the prognostic effects of signaling pathways based on the correlation between NES of pathways and clinical outcomes. We found the pathway, regulation of cell cycle phase transition, was a prognostic unfavorable pathway in IGC but was a prognostic favorable pathway in DGC. Conversely, the pathway, leukocyte aggregation, was a prognostic unfavorable pathway in DGC but was a prognostic favorable pathway in IGC. These results validated that cell cycle- and immune response-related signaling pathways were associated with opposite clinical outcomes between DGC and IGC (Supplementary Fig. 6c).

As cell cycle status impacted the sensitivity of patients to adjuvant chemotherapy^42^, we compared the prognoses of patients who underwent adjuvant chemotherapy and those who did not in each subtype. We found that DGC cluster 1 patients were insensitive to adjuvant chemotherapy, whereas IGC cluster 3 patients were sensitive (Fig. 4e, Supplementary Fig. 7c). To evaluate whether tumor cell cycle phases affected the sensitivity of patients to chemotherapy, we performed further statistical analysis and found that DGC cluster 1 had the highest percentage of patients with upregulated S phase signature proteins, whereas IGC cluster 3 had the highest percentage of patients with upregulated G2M phase transition signature proteins (Fisher’s exact test, *p* = 0.0128; Fig. 4f, Supplementary Data 5d). As reported, S and G2M phases were featured by DNA replication and cell division, respectively^43^. We further compared the key proteins involved in DNA replication and cell division, and found that these proteins had reverse expression patterns in DGC cluster 1 and IGC cluster 3 patients (two-sided Wilcoxon rank-sum test, *p* < 0.05, foldchange > 2; Fig. 4g, Supplementary Data 5e). Thus, chemotherapy treatment strategies should be devised after considering cell cycle phases of the tumor cells. We proposed proteins involved in DNA replication were worth to be considered as therapeutic targets for DGC cluster 1 (Fig. 4g).

Comparison of the DGC cluster 1 and IGC cluster 3 phospho-proteomes revealed the activation of subtype-specific kinases, such as CDK2 and CDK1, respectively (Fig. 4h). Further investigation into the differential phospho-sites showed that increase in CDK2 substrates and decrease in CDK1 substrates were observed in DGC cluster 1 (two-sided Wilcoxon rank-sum test, *p* < 0.05, foldchange > 2; Fig. 4g, Supplementary Data 5f). Survival analysis revealed CDK2 was associated with good prognosis in DGC and with poor prognosis in IGC (Log-rank test, *p* < 0.05; Supplementary Fig. 7d). These observations demonstrated that the activity of CDKs, particularly CDK1 and CDK2, associated with diverse prognoses among GC patients. As previously reported, CDK1 promoted G2-M transition, whereas CDK2 promoted DNA replication and S phase transition^43^. Our findings for DGC cluster 1 and IGC cluster 3 were consistent with these observations. As cell proliferation status and cell cycle phases affected a patient’s response to chemotherapy, we attempted to predict the chemotherapeutic response in GC patients based on CDK1 and CDK2 expression levels. In our GC cohort, we found that patients with high CDK1 and low CDK2 levels benefited from adjuvant chemotherapy (Log-rank test, *p* < 0.05; Fig. 4i), indicating that CDK1 and CDK2 levels could serve as biomarkers to gauge chemotherapeutic response in GC patients.

In summary, our proteomic subtypes showed the converse correlation between protein features and prognoses in DGC and IGC, which provided guidance for patient stratification and therapy strategies in clinic.

### TF activity profiles and their clinical relevance

Screening DNA-binding activity of TFs in GC can advance our understanding of GC heterogeneity. Despite the application of proteome profiling have made great progress in precision oncology, the existing strategies of quantifying the changes in TF activities have certain limitations^44^. The sub-proteome consisting of TFs is usually neglected in cancer proteomics because of low abundance of TFs. Therefore, we detected and evaluated inferred TF activities at proteomic level by TFRE approach which we previously developed^16,17^. We constructed the TF activity profiles for the 196 pairs of GC tumor tissues and NATs.

**Fig. 5:**
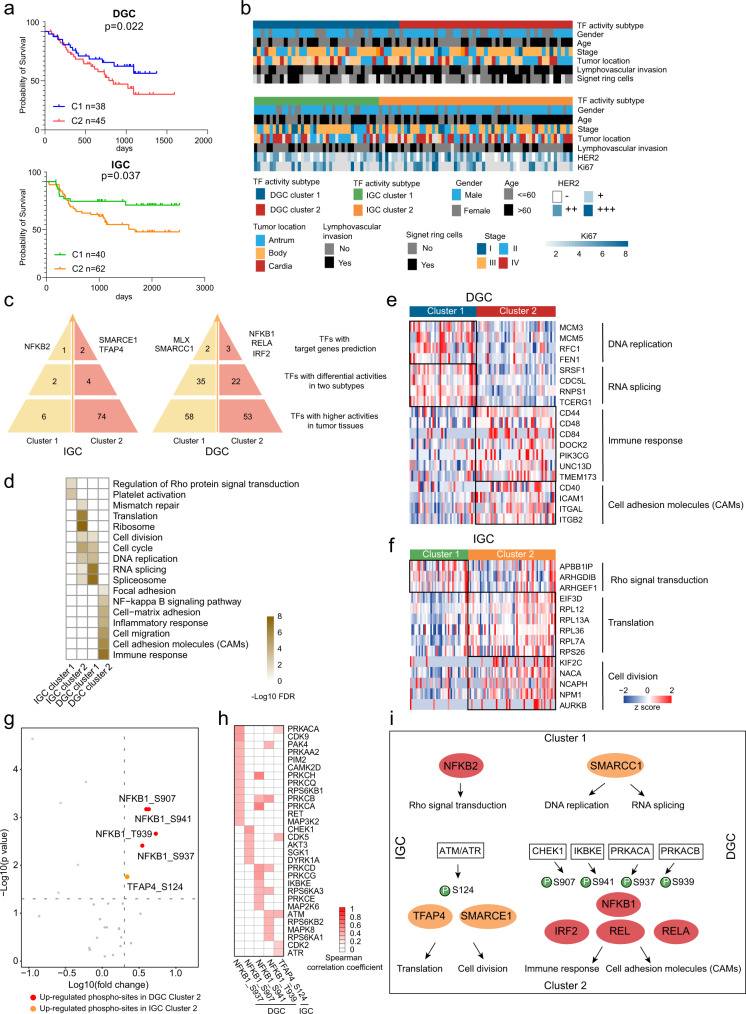
DGC and IGC subtypes based on TF activity profiles. **a** The association of TF activity-based subtypes with clinical outcomes in DGC and IGC. *n* (DGC cluster 1) = 38, *n* (DGC cluster 2) = 45, *n* (IGC cluster 1) = 40, and *n* (IGC cluster 2) = 62 biologically independent samples. *P*-values are from Log-rank test. **b** Clinical characteristics annotation in GC TF activity-based subtypes. **c** Master TFs selection in each TF activity-based subtype. **d** Pathway enrichment analysis of master TFs regulated TGs in each TF activity-based subtype. **e** A list of TGs regulated by master TFs in significantly altered pathways and their abundance in each DGC TF activity-based subtype. **f** A list of TGs regulated by master TFs in significantly altered pathways and their abundance in each IGC TF activity-based subtype. **g** Expression of phospho-sites in each TF activity-based subtype. The *p*-values are from Wilcoxon rank-sum test. Red and orange colors, upregulated phospho-sites in DGC cluster 2 and IGC cluster 2, respectively. **h** Spearman’s correlation coefficients between kinases and phospho-sites upregulated in DGC cluster 2 and IGC cluster 2. **i** Phospho-regulatory network in GC. Source data are provided as a Source Data file.

We performed cluster analysis of TF activity profiles of DGC and IGC with 425 and 396 TFs detected in >50% DGC and IGC patients, respectively, and identified two subtypes in each dataset (Supplementary Fig. 8a, b). Further analysis of the TF activity-based subtypes demonstrated their significant correlation with patients’ survival (Log-rank test, *p* < 0.05), indicating the prognostic power of clustering TF activity profiles (Fig. 5a). For convenience, TF activity-based subtypes were designated as DGC TF cluster 1 (*n* = 40), DGC TF cluster 2 (*n* = 43), IGC TF cluster 1 (*n* = 42), and IGC TF cluster 2 (*n* = 60), respectively. Evaluation of the clinical features of TF activity-based subtypes revealed that the DGC TF cluster 2 comprised more patients with lymphovascular invasion (55.3% in cluster 1 and 75.6% in cluster 2) and a higher probability of antrum (26.3% in cluster 1 and 46.7% in cluster 2) than DGC TF cluster 1 (Fig. 5b). The IGC TF cluster 2 comprised less stage I GC patients (2.6% in cluster 1 and 8.9% in cluster 2) than IGC TF cluster 1.

Subsequently, we identified the master TFs in each TF activity-based subtype (Fig. 5c, Supplementary Fig. 8c–e, Supplementary Data 6a). We found that NFKB2 dominated in IGC TF cluster 1; SMARCE1 and TFAP4 dominated in IGC TF cluster 2; MLX and SMARCC1 dominated in DGC TF cluster 1; NFKB1, RELA, and IRF2 dominated in DGC TF cluster 2. In total, NFKB complex was nominated as the master TFs in IGC TF cluster 1 and DGC TF cluster 2; SWI/SNF complex was nominated as the master TFs in IGC TF cluster 2 and DGC TF cluster 1. Notably, for DGC patients, DGC TF cluster 1 had better prognosis, whereas DGC TF cluster 2 had worse prognosis; for IGC patients, IGC TF cluster 1 had better prognosis, whereas IGC TF cluster 2 had worse prognosis (Log-rank test, *p* < 0.05). These results showed the diverse prognostic correlation of NFKB complex and SWI/SNF complex in DGC and IGC.

Pathway enrichment analysis of TGs demonstrated that the master TFs regulated different biological functions in different clusters (Fig. 5d, Supplementary Data 6b). For example, the NFKB complex involved in Rho protein signal transduction and platelet activation in IGC TF cluster 1, while it involved in immune response, CAMs translation, and cell migration in DGC TF cluster 2. On the other hand, the SWI/SNF complex involved in translation and cell cycle progression in IGC TF cluster 2, while it involved in RNA splicing and DNA replication in DGC TF cluster 1 (Fig. 5e–f).

A question that we posed was why master TFs could regulate a different set of genes in different subtypes. As phosphorylation is a fundamental mechanism to regulate TF activities, we explored the effect of phosphorylation on master TFs based on the kinase-substrate network. We compared the phosphorylation levels of these TFs and found that phosphorylation of NFKB1 at S907, S937, S939, and S941 were increased in DGC TF cluster 2, while phosphorylation of TFAP4 at S124 was increased in IGC TF cluster 2 (Wilcoxon rank-sum test, BH adjusted *p* < 0.05, foldchange > 2; Fig. 5g). Subsequently, we screened for kinases that were possibly responsible for these five phospho-sites by correlation analysis. We found 33 kinases had significant positive correlation with these five phospho-sites (spearman’s correlation coefficient > 0, *p* < 0.05; Fig. 5h, Supplementary Data 6c). The signal transduction network of TF activity-based subtypes was depicted in Fig. 5i. In DGC TF activity cluster 2, the kinase activity of IKBKE was correlated with phospho-site S941 of NFKB1. This indicated that IKBKE activated NFKB1, which was consistent with the previous studies^45^. In IGC, ATM/ATR activity had a significantly positive correlation with TFAP4 (phosphorylated at S124), which associated with the expression of cell division-related proteins. As shown in Fig. 3g, we found that ATM/ATR had higher activities in IGC than in DGC. These observations indicated a potential role of ATM/ATR in regulating cell division in DGC and IGC oncogenesis via the activation of distinct downstream TFs such as TFAP4. Here, we elucidated the roles of TF complexes in pathological processes and presented the kinase-TF-target gene network in DGC and IGC subtypes based on integrating multilevel proteomic data (Fig. 5i).

Additionally, we found the master TFs were correlated with prognoses among patients treated with adjuvant chemotherapy or not. For example, among patients with higher activity of SMARCC1 in IGC or lower activity of NFKB1 in DGC, who received adjuvant chemotherapy presented good prognosis (Log-rank test, *p* < 0.05). Thus, IGC patients with high SMARCC1 activity and DGC patients with low NFKB1 activity could benefit from chemotherapy (Supplementary Fig. 8f). Moreover, our findings concurred with previous reports that NFKB1 is involved in resistance to chemotherapy and radiotherapy^46^, indicating that NFKB1 and SMARCC1 could be potential biomarkers for GC diagnosis and for selection of an effective treatment strategy.

### Characteristics of multilevel proteomic subtyping and its robustness

**Fig. 6:**
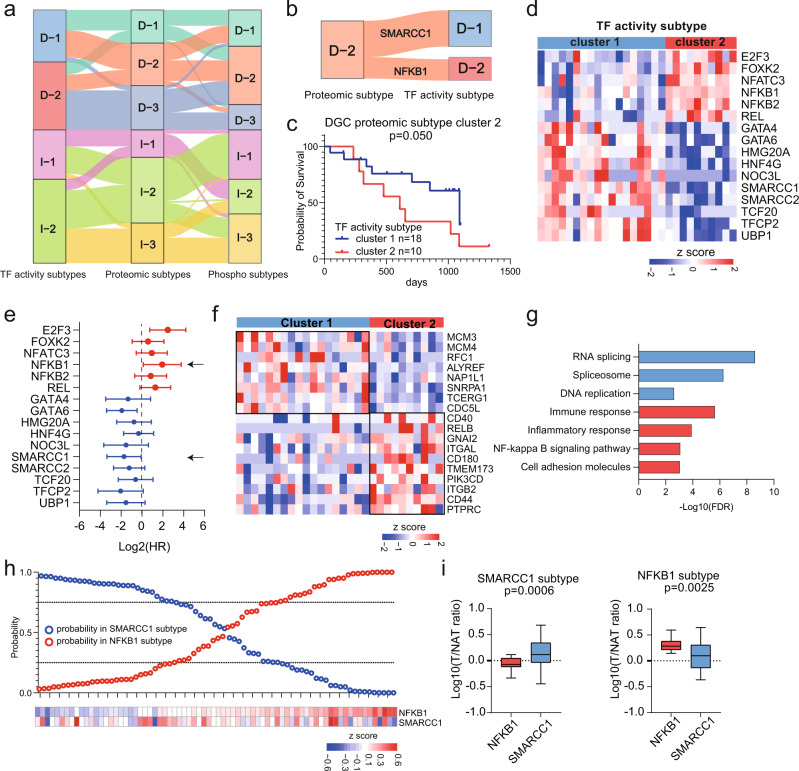
Characteristics of Multilevel Proteomic Subtyping and its Robustness. **a** Sankey diagram depicting the association of samples classified into TF activity, proteome and phospho-proteome-based subtypes. **b** Sankey diagram depicting the association of samples classified into DGC proteomic cluster 2 and DGC TF subtypes. **c** Prognostic outcomes of GC patients in DGC proteomic subtype cluster 2. *n* (cluster 1) = 18 and *n* (cluster 2) = 10 biologically independent samples. The *p*-value is from Log-rank test. **d** TF activities comparison between two TF activity subtypes. **e** Prognostic outcomes of TFs with significantly differential activities in two TF activity subtypes. *n* = 28 biologically independent samples. The points and error bars show the median of hazard ratio (HR) and 95% confidence interval (CI). **f** Proteins expression of target genes of two TFs. **g** Pathways enriched in two subgroups of DGC proteomic subtype cluster 2. **h** Performance of the TF subtype predictor based on NFKB1 and SMARCC1. **i** The expression of NFKB1 and SMARCC1 in two subgroups. *n* (SMARCC1 subtype)  = 28 and *n* (NFKB1 subtype) = 24 biologically independent samples. Boxplots show median (central line), upper and lower quartiles (box limits), min to max range. The *p*-value is calculated using two-sided student’s *t*-test. Source data are provided as a Source Data file.

We performed consensus clustering analysis based on phospho-proteomic data. We applied phospho-sites detected in >50% DGC and IGC patients, corresponding to 4484 and 4739 phospho-proteins, respectively, in consensus clustering and identified three DGC phospho-proteomic subtypes and three IGC phospho-proteomic subtypes. We designated the subtypes as DGC phospho-proteomic cluster 1 (*n* = 27), DGC phospho-proteomic cluster 2 (*n* = 37), and DGC phospho-proteomic cluster 3 (*n* = 16) in DGC; and IGC phospho-proteomic cluster 1 (*n* = 27), IGC phospho-proteomic cluster 2 (*n* = 26), and IGC phospho-proteomic cluster 3 (*n* = 30) in IGC, respectively (Supplementary Fig. 9). Then, we summarized these subtyping results from individual dataset. As shown in Supplementary Table 2, except correspondence between phospho-proteomic subtypes and TF activity-based subtypes in IGC, the statistical results of classification concordance among subtypes based on three datasets were all significant (chi-square test, *p* < 0.05). These results demonstrated that our TF activity-based subtypes, proteomic subtypes and phospho-proteomic subtypes had high classification concordance (Fig. 6a).

To explore the characteristics of each phospho-proteomic subtype and correspondences between proteomic subtypes and phospho-proteomic subtypes, differentially expressed phospho-sites (Wilcoxon rank-sum test, BH adjusted *p* < 0.05, foldchange > 2) were identified, and pathway enrichment analysis was performed. As shown in Supplementary Fig. 9b, c, DGC phospho-proteomic cluster 1 was characterized by RNA splicing, cell cycle, DNA repair and RHO GTPase cycle, which were corresponding to characteristics of DGC proteomic subtypes cluster 1; DGC phospho-proteomic cluster 2 was characterized by cytoskeleton organization, which showed similar features with DGC proteomic cluster 2; DGC phospho-proteomic cluster 3 was characterized by cadherin binding and cell adhesion molecule binding, which showed similar features with DGC proteomic cluster 3; IGC phospho-proteomic cluster 1 was characterized by cytoskeleton organization and actin cytoskeleton organization, which were corresponding to characteristics of proteomic subtype cluster 2; IGC phospho-proteomic cluster 2 was characterized by RNA splicing and DNA repair; and IGC phospho-proteomic cluster 3 was characterized by cell cycle, which was corresponding to characteristics of proteomic subtype cluster 3.

In addition, we explored the association between TF activity-based subtypes and proteomic subtypes. We found that 65% (15 out of 23) DGC proteomic subtype cluster1 comprised a subset of the DGC TF activity subtype cluster1; 96% (27 out of 28) DGC proteomic subtype cluster 3 comprised a subset of the DGC TF activity subtype cluster 2; 61% (11 out of 18) IGC proteomic subtype cluster1 comprised a subset of the IGC TF activity subtype cluster 1; 84% (21 out of 25) IGC proteomic subtype cluster 3 comprised a subset of the IGC TF activity subtype cluster 2. These results showed that the proteomic subtypes were significantly overlapped with the TF activity subtypes. Interestingly, patients in DGC proteomic subtype cluster 2 were grouped into two TF activity subtypes (18 patients (64%) in TF activity subtypes cluster 1 and 10 patients (36%) in TF activity subtypes cluster 2, Fig. 6b).

For DGC proteomic subtype cluster 2, we further explored the clinical and molecular differences between two TF activity-based subtypes. As expected, patients in TF activity subtype cluster 2 had worse prognosis (Fig. 6c), featured by higher TF activity of NFKB1 and lower TF activity of SMARCC1 (Fig. 6d). Further prognostic analysis of TF activities revealed that TF activity of NFKB1 was negatively correlated with the prognosis, and the TF activity of SMARCC1 showed positive correlation with the prognosis (Fig. 6e). Accordingly, combination of these two TF activities could well distinguish patients with poor prognosis (with high activity of NFKB1 and low activity of SMARCC1) from those with good prognosis (with high activity of SMARCC1 and low activity of NFKB1), exhibiting good prognostic predictive capacity (Supplementary Fig. 10a). At last, we compared the expression of TGs of NFKB1 and SMARCC1 based on proteomic dataset (Fig. 6f, g). We found SMARCC1’s target genes, involved in RNA splicing and DNA replication, were upregulated in TF activity subtype cluster 1; NFKB1’s target genes, related to immune response, were upregulated in TF activity subtype cluster 2. These results were consistent with pathway enrichment in DGC phospho-proteomic subtype cluster 1 and cluster 2 (Supplementary Fig. 10b). Overall, integrated subtyping results suggested that proteomic subtypes coupled with TF activity analysis could be exploited for prognostic prediction and combinatorial therapeutic strategy.

To validate the robustness of the proteomic subtyping, the Mun’s cohort^10^, which was subtyped into Prot 1 (immune response related processes), Prot 2 (actin cytoskeleton and cadherin signaling), Prot 3 (metabolism), and Prot 4 (RNA processing), was used as an independent validation cohort. Based on the 200 most representative proteins of each proteomic subtype identified in our cohort, the Mun’s cohort were reanalyzed and clustered into three proteomic subtypes: subtype 1 (*n* = 23), subtype 2 (*n* = 24), and subtype 3 (*n* = 27) (Supplementary Fig. 10b, Supplementary Data 6d). The signature proteins and subtype-specific pathways (subtype 1: spliceosome, corresponding to Prot 3 and 4; subtype 2: ECM organization, corresponding to Prot 2; and subtype 3, immune response, corresponding to Prot 1) were shown in Supplementary Fig. 10c–e. We performed chi-square test to assess the classification concordance between proteomic subtypes and Mun’s subtypes. The statistical results of classification concordance were significant (chi-square test, *p* < 0.05, Supplementary Fig. 10f). The high classification concordance demonstrated that the consistent expression pattern of signature proteins dominant in our subtyping could be observed in Mun’s cohort, supporting the reliability of our subtyping.

To further validate the classification power of TF activity, we used a Bayesian algorithm to distinguish patients in Mun’s cohort into two TF subtypes (NFKB1 subtype and SMARCC1 subtype)^47^. In Mun’s cohort, 24 and 28 cases were identified as NFKB1 subgroup and SMARCC1 subgroup, respectively (Fig. 6h, i). As shown in Supplementary Fig. 10e, we found that the patients in subtype 2 of proteomic subtypes were assessed as two TF activity subtypes (9 patients in SMARCC1 subtype and 8 patients in NFKB1 subtype) in Mun’s cohort. We observed the similar corresponding association of TF activity subtypes and proteomic subtype cluster 2 in our cohort and Mun’s cohort. Also, statistical analysis showed the classification concordance between proteomic subtypes and TF subtypes of Mun’s cohort were significant (chi-square test, *p* < 0.05, Supplementary Fig. 10f). These results showed that our TF activity subtypes were robust, which could be supported by the published GC dataset.

### Immune characterization of GC tumors

Tumor microenvironment (TME) comprises tumor cells, cancer-associated fibroblasts, infiltrating immune cells, and endothelial cells^18^. Several studies have indicated that the TME influences cancer progression and therapeutic responses in patients^19^. Although recent advances in immunotherapy and targeted drug therapy in treating GC patients have improved patient prognosis, these therapies are efficient only for a subset of patients. It is imperative to address indicators for immunotherapeutic effectiveness.

**Fig. 7:**
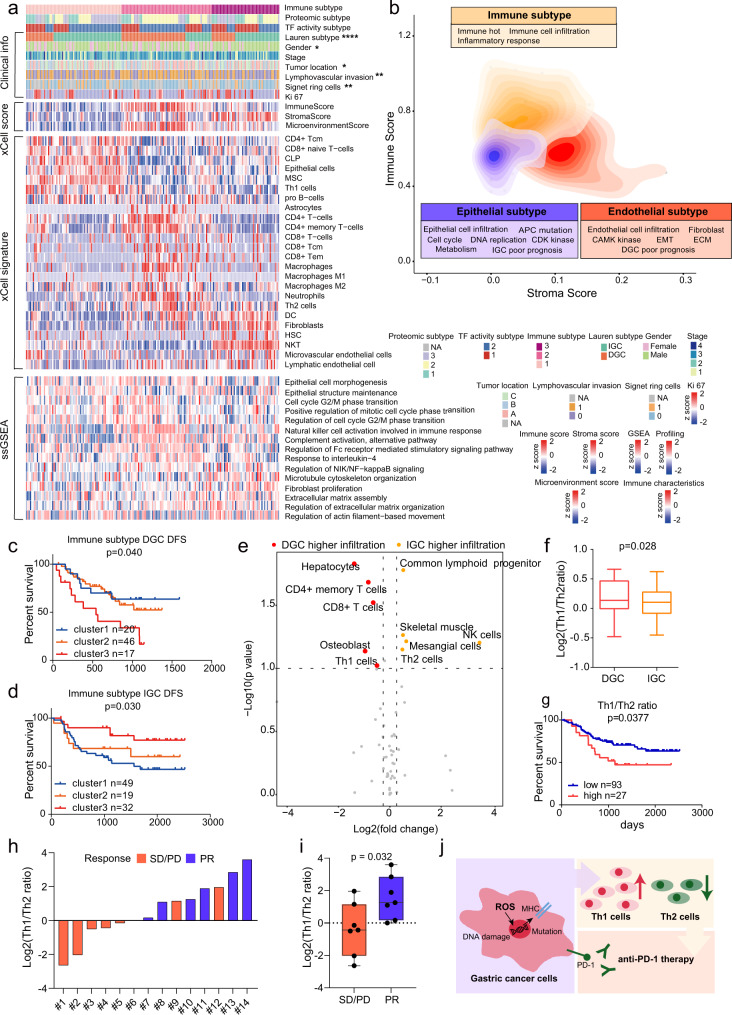
Characterization of immune infiltration in GC. **a** Heatmap illustrating the immune/stroma signatures from xCell, and ssGSEA pathway scores in each immune subtype. *P*-values are from two-sided chi-square test. The *p*-values are 1.64E-6 (Lauren subtype), 0.017(Gender), 0.027(Tumor location), 0.01(Lymphovascular invasion), and 0.0059(Signet ring cells). **b** Contour plot of two-dimensional density based on immune score (*y*-axis) and stroma score (*x*-axis) among different immune clusters. **c**, **d** Kaplan–Meier curves of DFS for DGC and IGC based on immune subtypes. *n* (DGC cluster 1) = 20, *n* (DGC cluster 2) = 46, *n* (DGC cluster 3) = 17, *n* (IGC cluster 1) = 49, *n* (IGC cluster 2) = 19, and *n* (IGC cluster 3) = 32 biologically independent samples. *P*-values are from Log-rank test. **e** Immune cell infiltration between DGC and IGC. The *p*-values are from two-sided Wilcoxon rank-sum test. **f** Th1/Th2 ratio in DGC and IGC. *n* (DGC) = 17 and *n* (IGC) = 32 biologically independent samples. Boxplots show median (central line), upper and lower quartiles (box limits), min to max range. *P*-values are calculated using two-sided student’s *t*-test. **g** The association of Th1/Th2 ratio with prognostic outcomes in all GC patients. *n* (low) = 93 and *n* (high) = 27 biologically independent samples. *P*-values are from Log-rank test. **h** Distribution of Th1/Th2 ratio in the GC anti-PD-1 patient group. **i** Comparison of Th1/Th2 ratio between responder and non-responder groups. *n* (PR) = 7 and *n* (SD/PD) = 7 biologically independent samples. Boxplots show median (central line), upper and lower quartiles (box limits), min to max range. *P*-values are calculated using two-sided student’s *t*-test. Each point represents a sample. **j** Summary of T helper cells recruitment mechanism in GC. ****p < 1.0e-4, ***p < 1.0e-3, **p < 0.01, *p < 0.05. Source data are provided as a Source Data file.

To better understand the concept of immune cell infiltration in GC tumors, we performed xCell^35^ analysis of the proteomic data to infer the relative abundance of diverse cell types in the TME (Fig. 7, Supplementary Fig. 11). Consensus clustering based on inferred cell proportion identified the following three sets of tumors with distinct immune signatures and stromal features: immune cluster 1 (*n* = 69), immune cluster 2 (*n* = 65), and immune cluster 3 (*n* = 49; Fig. 7a, b, Supplementary Fig. 11a). We found that immune cluster 1 had lower immune and stoma scores (ANOVA, *p* < 0.001) and had a higher proportion of epithelial cells than other clusters. As expected, ssGSEA analysis indicated the epithelial cell morphogenesis and positive regulation of mitotic cell cycle phase transition were elevated in immune cluster 1. Furthermore, canonical markers of epithelial cells, i.e., EPCAM, KRT18, MUC1, and CDH1 had the highest expression in immune cluster 1 than other clusters (Fig. 7a, Supplementary Fig. 11b–d). Immune cluster 2 had higher immune and microenvironment scores (two-sided ANOVA, *p* < 0.001), higher proportions of the CD4 + T cells, neutrophils, and macrophages, and lower proportion of the natural killer T cells than other clusters. This observation might be supported by that pathways such as complement activation and regulation of NIK/NFKB signaling were elevated in immune cluster 2. Moreover, canonical markers of macrophages, i.e., TLR2 and ARG1^48^, and immunotherapeutic targets, i.e., FCGR1A, CD276, and CD27^20^, had higher expression in immune cluster 2 than other clusters (Fig. 7a, Supplementary Fig. 11b–e, Supplementary Data 7a). As for immune cluster 3, we observed higher stroma score (two-sided ANOVA ANOVA, *p* < 0.001) and higher proportion of fibroblasts, lymphatic endothelial cells, and microvascular endothelial cells than other clusters. Fibroblast proliferation, ECM assembly and regulation of actin filament-based movement were enriched in immune cluster 3. The canonical marker of endothelial cells, DCN, had higher expression in immune cluster 3 than other clusters (Fig. 7a, Supplementary Fig. 11b–d). Thus, the immune subtypes were defined as epithelial subtype (cluster 1, cold tumor), immune subtype (cluster 2, hot tumor), and endothelial subtype (cluster 3, Fig. 7b).

Multivariate cox regression analysis revealed immune subtypes (cluster 1–3) were associated with prognoses after adjusting for other clinical covariates (Log-rank test, *p* < 0.05; Fig. 7c, d). Interestingly, we found that DGC and IGC patients in immune cluster 3 exhibited a opposite prognostic trend. For IGC patients, immune cluster 3 had the best prognosis, while for DGC patients, immune cluster 3 had the worst prognosis (the red lines; Fig. 7c, d). To address this issue, we compared tumor infiltration of immune cells in DGC and IGC patients in immune cluster 3. The common lymphoid progenitor, NK cells, and Th2 cells exhibited higher levels in IGC patients than in DGC patients, whereas CD4 + memory T cells, CD8 + T cells, and Th1 cells exhibited higher levels in DGC patients than in IGC patients (Wilcoxon rank-sum test, *p* < 0.1, foldchange > 1.2; Fig. 7e, Supplementary Data 7b). DGC patients had a higher Th1/Th2 ratio than IGC patients in immune cluster 3 (Fig. 7f). It has been previously reported that Th1/Th2 ratio could be used as a prognostic marker^49^. Moreover, Mohammadi et al. demonstrated that Th1 and Th2 cells had differential contribution with respect to immune response to *Helicobacter pylori* infection-related gastritis. The Th1 cells were involved in pathogenesis, and the Th2 cells were associated with protection from the infection^49^. Th2 cells, not Th1 cells, reduced inflammation and showed beneficial effects on GC treatment^50^. Significantly, in our cohort, Th1/Th2 ratio was negatively associated with prognosis for all GC patients (Log-rank test, *p* = 0.0377; Fig. 7g), demonstrating that the Th1/Th2 ratio could serve as a prognostic indicator in GC patients. To validate this conclusion, the Th1/Th2 ratio values were calculated by evaluation of xCell based on TCGA transcriptomic dataset^7^. We compared the Th1/Th2 ratio between DGC and IGC, and analyzed the association of Th1/Th2 ratio with prognosis. We found the ratio of Th1/Th2 was higher in DGC than IGC, and this ratio value was negatively related to the prognosis in TCGA cohort (Supplementary Fig. 12a, b). These results were consistent with the results observed in our proteomic data, which validated the prognostic effect of Th1/Th2 ratio in GC.

We further explored the reason as to why there was higher tumor infiltration by Th1 cells in DGC than in IGC. We calculated the spearman’s correlation coefficients for Th1 cell scores and GSEA pathway NESs. We found that generation of reactive oxygen species (ROS) had the highest correlation with Th1 cells (spearman’s correlation coefficient = 0.72, *p* = 0.0012; Supplementary Fig. 12c). This indicated that ROS may affect Th1 cell recruitment in DGC. Notably, ROS generation is one of the hallmarks of cancer progression, and causes oxidative damage to DNA, proteins and lipids^51^. Furthermore, ROS increases the mutational load and enhances antigen processing and presentation, which was a common mechanism that affects the immune microenvironment^52^. Subsequently, correlation analysis of pathway NESs revealed that ROS generation, cellular response to DNA damage, mutational load, and antigen processing and presentation via MHC class II were significantly and positively correlated with each other (Supplementary Fig. 12d). We found a significantly positive correlation (spearman’s correlation coefficient = 0.77, *p* = 0.025) between the Th1/Th2 ratio and mutational load in immune cluster three patients (Supplementary Fig. 12e). These results reported a potential mechanism that elevated ROS in DGC increased the expression of MHC class II molecules in response to DNA damage and mutational load increase, subsequently recruiting more Th1 cells (Supplementary Fig. 12f).

To validate the correlation between Th1/Th2 ratio and immunotherapeutic effectiveness, we collected a group of GC patients treated with anti-PD1 therapy, including 7 responder cases (PR) and 7 non-responder cases (SD/PD) (Supplementary Data 7c). The formalin-fixed paraffin-embedded (FFPE) tumor tissue sections derived from 14 therapy-naïve GC patients were collected. Proteomics measurement resulted in 7705 proteins in total. On an average, 4575 proteins were identified per sample. The immune cells infiltration in the 14 samples were evaluated by xCell analysis based on the proteomic profiles. The Th1/Th2 ratio values of 14 samples were calculated as shown in Fig. 7h. We found that the Th1/Th2 ratio was significantly higher in the responder group compared to the non-responder group (Fig. 7i). This result suggested that the Th1/Th2 ratio could be an indicator for predicting clinical outcomes of immunotherapy among GC patients (Fig. 7j). Therefore, the relationship between Th1/Th2 ratio and immunotherapeutic effectiveness was further validated in an independent gastric cancer anti-PD1 therapeutic patient group.

## Discussion

GC is one of the main cancer types worldwide; the global 5-year survival rates for GC patients remain ~25–30%^4^. In clinical diagnosis, Lauren classification is used for the preliminary diagnosis of GC patients. However, molecular characteristics of Lauren classification (DGC and IGC in major) are unclear, which hinder appropriate treatment approaches application for patients with different pathologies. In this study, we constructed a multilevel proteomic landscape by analyzing the proteome, phospho-proteome, and TF activity profile datasets. TFRE approach^16,17^, a DNA pull-down-based TF activity assay, was used in this study to infer the activity of TFs. TFRE approach could detect and quantify more TFs than proteome, which provided more detailed proteomic landscape. The integrated analysis among TF activity profile and proteome constructed the TF-TG signal transduction network, which provided biological mechanisms of tumor processes and potential drug targets. The proteome, phospho-proteome, and TF activity profile provided insights into the biological processes underlying GC, from protein abundance, post-translational modification to TF activity, indicating the importance of our GC protein landscape. Phospho-proteome and TF activity profile increased the identifications of kinases and TFs in tryptic peptides samples, allowing us to compare the results more deeply in proteomic analyses. This study focused on quantification analyses within platforms, while the comparison among different platforms is also an important issue need to further study.

Multilevel proteomic analysis indicated that DGC and IGC were associated with different prognoses and pathogenic mechanisms, thus, requiring different therapeutic options. We found that DNA damage was upregulated in IGC, whereas immune and ECM proteins were upregulated in DGC. It is possible that ATM/ATR, the key kinases in DNA mismatch repair, regulated cell proliferation in IGC by activating the SWI/SNF complex. Therefore, we proposed ATM/ATR as potential therapeutic targets for IGC. The potential targets for treating DGC are CDK4/6, which regulated cell cycle in DGC by activating the RB1/E2F pathway^53^. Analysis of TCGA data revealed that 66% of the GC patients exhibited altered expression of at least one of the following cell cycle related genes: *RB1*, *CCND1*, *CCNE1*, *CDK2*, *CDK4*, *CDK6*, *CDKN2A*, *CDKN2B*, *E2F1*, *E2F2*, *E2F3*, and *E2F4*^54^. Moreover, molecular dissection of the chromosome band 7q21 amplicon in gastroesophageal junction adenocarcinomas revealed upregulated CDK6 expression at both transcription and translation levels^54^. Targeting CDK4/6 have been reported to improve patient outcomes in clinical trials in a variety of tumor types^53^, which are also worth to investigate in GC. Besides in our cohort, we validated in TCGA cohort that CDK4/6 and ATM/ATR were the potential targets for DGC and IGC, respectively. These results indicated the universality of this conclusion, suggesting these potential targets need to be further tested in clinical trial.

Our proteomic and TF activity-based subtypes showed the reverse correlation between protein/TF features and prognoses in DGC and IGC. The validation of subtypes in an independent cohort illustrated the robustness of our subtypes. The aberrations in biological processes among subtypes provided guidance for patient stratification and therapy strategies in clinic. Based on the analysis of proteomic subtypes, we presumed that analyzing the cell cycle phases could improve the chemotherapeutic efficacy, and CDK1/2 could be used as biomarkers predicting chemotherapeutic response. TF activity-based subtypes showed the importance of TFs SMARCC1 and NFKB1 in DGC and IGC. The SWI/SNF chromatin remodeling complex controls stemness, differentiation, and proliferation, etc^13^. NFKB complex was reported to play important roles in immune responses, cell proliferation, cell death, and inflammation, etc^55^. Nevertheless, it is difficult for TFs to be targeted by small molecule inhibitors as they lack functional sites or allosteric regulatory pockets that generally exist in kinases or other enzymes. In addition to development of agents to inhibit cytoplasmic proteases that activate NFKB, direct approaches such as Proteolysis Targeting Chimeras (PROTACs) are emerging^56^. These treatment approaches can be employed to treat patients with DGC TF cluster 2 subtype.

We performed immune subtyping based on the inferred immune cell scores and defined three immune subtypes (epithelial subtype, immune subtype, and endothelial subtype). Characteristics extraction and pathway enrichment analysis suggested TME involved molecular regulation mechanisms. For example, we proposed that tumor infiltration by immune cells, such as macrophages, were associated with metastasis by activating the NFKB complex in DGC. Prognostic analysis of immune subtypes proved that DGC and IGC patients in immune cluster 3 exhibited reverse prognostic association. Furthermore, we found that the Th1/Th2 ratio was differential in DGC and IGC, and this value could serve as an indicator to predict immunotherapeutic effectiveness. This result was validated in published TCGA cohort and an anti-PD1 immunotherapeutic patient group. Additionally, our data indicated that the recruitment of T helper cells was linked to ROS level and mutational load. We observed patients with high Th1/Th2 ratio, who responded to anti-PD1 therapy, had highly expressed inducers and lowly expressed scavengers of ROS. These results indicated that immunotherapy responders had higher Th1/Th2 ratio and increased ROS level. Antioxidant therapy, which depresses the level of ROS by antioxidants, has been reported to improve clinical outcomes of tumor patients^52^. We believe that Th1/Th2 ratio could serve as a biomarker to determine the selection of antioxidant therapy for GC patients, which required further investigation.

In summary, our research performed comprehensive proteomic analyses of DGC and IGC. Multilevel proteomic subtypes were identified with distinct molecular features and clinical outcomes.

## Methods

### The construction of the GC cohort

The Medical Research Ethics Committees of Peking University Cancer Hospital (2015KT70), Xijing Hospital (KY20150415), Chinese PLA General Hospital (S2016-057-02), and Zhongshan Hospital (B2019-200R) approved this study, and all patients provided written informed consent for sample collection, analysis, and publishing basic and clinicopathological information.

We selected 83 cases of diffuse-type gastric cancer (DGC), 102 cases of intestinal-type gastric cancer (IGC) and 11 cases of mixed gastric cancer (MGC) from Peking University Cancer Hospital, Xijing Hospital, and Chinese PLA General Hospital. These 196 patients underwent total or subtotal gastrectomy between 2012 and 2015, and no patient in this cohort was treated with neoadjuvant chemotherapy or chemo-radiation therapy before operation. The surgical treatments were performed by clinicians according to guidelines^57^. All cases were staged according to the seventh edition of the American Joint Committee on Cancer (AJCC) staging system. The corresponding NATs were selected 5 cm away from the sites at which the primary tumor tissues were sampled. The muscle layers were carefully removed using a scalpel and fine forcep, and the mucosa layers were used as NATs. Each specimen was collected within 30 min after operation, cleaned with sterile towel, immediately transferred into sterile freezing vials and immersed in liquid nitrogen, then stored at −80 °C until use. Tumor tissues and their nearby tissues were evaluated by pathologists. Specimens in dry ice were transferred to National Center for Protein Sciences (The PHOENIX Center, Beijing).

The date of operation was used as a surrogate for the date of initial diagnosis. Overall survival (OS) was defined as the interval between the date of initial surgical resection to the date of last known contact or death. Disease free survival (DFS) was defined as the interval between the date of initial surgical resection to the date of progression or to the last follow-up date. There were 144 patients (~75%) received chemotherapy after surgery. Whether patients receive chemotherapy or not was based on the clinical guidelines, patients’ prognosis and the patients’ willingness. With or without chemotherapy in this research was defined as with or without at least one cycle of adjuvant chemotherapy. Demographics, histopathologic information, primary tumor location, treatment details including chemotherapy drugs, doses and routes of administration, and outcome parameters were collected. Signet ring cell proportion, lymphovascular invasion, and Ki67 were also determined.

### Sample collection of the anti-PD1 patient group

We surveyed medical records of GC patients in the Department of Pathology, Zhongshan Hospital, Fudan University (Shanghai, R. P. China), and then screened 14 GC patients treated with anti-PD1 immunotherapy after surgery from December 2018 to August 2021. The treatment response was evaluated by CT/MRI scanning following the Response Evaluation Criteria in Solid Tumors (RECIST) (version1.1). Tumor response was assessed and categorized as a complete response (CR), partial response (PR), stable disease (SD), or progressive disease (PD). Here, patients with CR and PR were defined as responder and those with SD and PD were defined as non-responder. In the anti-PD1 patient group, 7 responders (PR) and 7 non-responders (SD/PD) were included. Detailed clinical information of each patient was included in Supplementary Data 7c. The formalin-fixed paraffin-embedded (FFPE) tissue sections derived from 14 therapy-naïve GC patients were collected, and the tumor regions were determined by pathological examination.

### Cell line

Human HEK293T (Cat# CRL-11268 from ATCC; RRID: CVCL_QW54) was obtained and cultured in DMEM (GIBCO) with 10% FBS (GIBCO) in 5% CO_2_ at 37 °C. Cells validation using short tandem repeat markers (STR) were performed by Meixuan Biological Science and Technology Ltd. (Shanghai). In detail, these cell lines were firstly tested cell species by PCR method using extracted total genomic DNA, and examined by STR profiling. Then, STR data were analyzed using the DSMZ (German Collection of Microorganisms and Cell Cultures) online STR database (http://www.dsmz.de/fp/cgi-bin/str.html). Cell lines were tested negative for mycoplasma contamination.

### Targeted exome sequencing

A capture panel was developed, which covered coding exons and flanking splicing junctions for 274 gastric cancer driver genes^9^. For each pair of tumor and paired NAT samples, genomic DNA was extracted using the Gentra Puregene (Qiagen). Briefly, 1 μg of genomic DNA from each sample was mechanically sheared, end repaired, and ligated to molecularly bar-coded adapters to generate sequencing libraries following the manufacturer’s standard protocol (Illumina). Captured sample DNA was sequenced on an Illumina HiSeq 2000 according to the standard operating protocol.

### Protein extraction and trypsin digestion

Samples were minced and lysed in lysis buffer (8 M urea, 100 mM Tris hydrochloride, pH 8.0) containing protease and phosphatase inhibitors (Thermo Scientific) followed by 1 min of sonication (3 s on and 3 s off, amplitude 25%). The lysate was centrifuged at 14,000 g for 10 min and the supernatant was collected as whole tissue extract. Protein concentration was determined by Bradford protein assay. Extracts from each sample (100 μg proteins) was reduced with 10 mM dithiothreitol at 56 °C for 30 min and alkylated with 10 mM iodoacetamide at room temperature (RT) in the dark for additional 30 min. Samples were then digested using the filter aided proteome preparation (FASP) method^58^ with trypsin. Briefly, samples were transferred into a 30kD Microcon filter (Millipore) and centrifuged at 14,000 g for 20 min. The precipitate on the filter was washed twice by adding 300 μL washing buffer (8 M urea in 100 mM Tris, pH 8.0) into the filter and centrifuged at 14,000 g for 20 min. The precipitate was resuspended in 200 μL 100 mM NH_4_HCO_3_. Trypsin with a protein-to-enzyme ratio of 50:1 (w/w) was added into the filter. Proteins were digested at 37 °C for 16 h. After tryptic digestion, peptides were collected by centrifugation at 14,000 g for 20 min and dried in a vacuum concentrator (Thermo Scientific).

Tryptic peptides were separated in a home-made reverse-phase C18 column in a pipet tip. Peptides were eluted and separated into nine fractions using a stepwise gradient of increasing acetonitrile (6%, 9%, 12%, 15%, 18%, 21%, 25%, 30%, and 35%) at pH 10. Nine fractions were combined to six fractions, dried in a vacuum concentrator (Thermo Scientific), and then analyzed by liquid chromatography tandem mass spectrometry (LC-MS/MS).

For FFPE sample preparation, sections (10 μm thick) from FFPE blocks were macro-dissected, deparaffinized with xylene, and washed with ethanol. The ethanol was removed completely and the sections were left to air-dry. FFPE samples were added lysis buffer [0.1 M Tris-HCl (pH 8.0), 0.1 M DTT (Sigma, 43815), 1 mM PMSF (Amresco, M145)] and lysed with 4% sodium dodecyl sulfate (SDS). The extracted solution was collected, and then added the pre-cold acetone with 4-fold volume. Subsequently, the acetone-precipitated proteins were washed with cooled acetone. Filter-aided sample preparation (FASP) procedure^58^ was used for protein digestion.

### Phospho-peptide enrichment

Tryptic peptides were used for phospho-peptide enrichment. 15 mg TiO_2_-coupled beads were incubated with 500 μl Binding buffer (BB) for 10 min. Separated TiO_2_ into three 1.5 mL EP tubes equally, 5 mg for each and centrifuged 2000 g for 2 min. Peptides were resolved with 100 uL BB solution and combined with 5 mg incubated TiO_2_ for 30 min. Then centrifuged 1000 g for 2 min to collect supernatant and transferred them to a second EP tube which included TiO_2_. Repeated the phospho-peptides procedure twice and then discarded the supernatant. TiO_2_ was washed with BB solution for five times. An additional washing procedure was carried out with the wash buffer 1 (30% ACN, 0.5% trifluoroacetic acid) for one time and then with the wash buffer 2 (80% ACN, 0.5% trifluoroacetic acid) for two times to further remove the unphosphorylated peptides. Peptides were eluted and separated into 6 fractions using a stepwise gradient of increasing acetonitrile (0%, 2%, 5%, 8%, 10%, 40%) at pH 10. Six fractions were combined into 3 fractions, dried in a vacuum concentrator (Thermo Scientific) and then analyzed by LC-MS/MS.

### Nuclear proteins extraction

The tissues were washed twice with ice-cold phosphate-buffered saline to remove blood and other contaminates, then suspended in 800 μL of Cytoplasmic Extraction Reagent I (CER I) buffer (NE-PER kit, Thermo Scientific) and homogenized using a tissue grinder. Nuclear proteins were extracted in accordance with the manufacturer’s instructions^59^. Protein concentrations were determined using the Bradford method. Approximately, 1 mg of the nuclear protein was extracted from each tissue sample.

### TFRE pull-down and trypsin digestion

DNA was synthesized by Genscript (Nanjing, Jiangsu Province, China). Biotinylated TFRE primers (Forward primer: 5'-CATTCAGGCTGCGCAACTGTTG-3', Reverse primer: 5'-GTGAGTTAGCTCACTCATTAGG-3') were synthesized by Sigma. Dynabeads (M-280 streptavidin) were purchased from Invitrogen. Approximately 2–3 pmol of biotinylated DNA was pre-immobilized on Dynabeads and then mixed with nuclear extracts (NEs) from the tissues. The mixtures were incubated for 2 h at 4 °C. The supernatant was discarded, and the Dynabeads were washed twice with NETN solution (100 mM NaCl, 20 mM Tris-HCl, 0.5 mM ethylenediaminetetraacetic acid and 0.5% (vol/vol) Nonidet P-40) and then twice with phosphate-buffered saline. The TFRE pull-down beads were resuspended with 20 μL of SDS loading buffer and boiled for 5 min at 95 °C. The samples were then loaded on 10 cm 10% SDS-polyacrylamide gel electrophoresis gels and run to 1/3 of the length. The gel was stained with coomassie brilliant blue and then destained in 5% ethanol/10% acetic acid solution. Six bands were excised according to the molecular weight ranges and then subjected to in-gel trypsin digestion. 0.1% formic acid was used to stop digestion and 50% acetonitrile was used to extract peptides. Peptide solution was dried in a vacuum concentrator (Thermo Scientific) and then analyzed by LC-MS/MS.

### LC-MS/MS analysis

The three kinds of peptide samples (proteome, phospho-proteome, and TF activity profile) were detected by Orbitrap analyzer-based mass spectrometers platforms. The proteomic peptide samples were detected on Orbitrap Fusion (Thermo Fisher Scientific, Rockford, IL, USA) mass spectrometers, the phospho-proteomic peptide samples were detected on Fusion Lumos mass spectrometers (Thermo Fisher Scientific, Rockford, IL, USA), and the TF activity profile peptide samples were detected on Q Exactive HF (Thermo Fisher Scientific, Rockford, IL, USA) mass spectrometers. Each layer dataset was acquired by the same mass spectrometer.

Dried peptide samples were re-dissolved in Solvent A (0.1% formic acid in water) and loaded to a trap column (100 μm × 2 cm, home-made; particle size, 3 μm; pore size, 120 Å; SunChrom, USA) with a max pressure of 280 bar using Solvent A, then separated on a home-made 150 μm × 12 cm silica microcolumn (particle size, 1.9 μm; pore size, 120 Å; SunChrom, USA) with a gradient of 5–35% mobile phase B (acetonitrile and 0.1% formic acid) at a flow rate of 350 nL/min for 75 min.

The eluted peptides were ionized under 2 kV. MS was operated under a data-dependent acquisition (DDA) mode. For detection with Fusion or Fusion Lumos mass spectrometer, a precursor scan was carried out in the Orbitrap by scanning m/z 300–1400 with a resolution of 120,000 at 200 m/z. The most intense ions selected under top-speed mode were isolated in Quadrupole with a 1.6 m/z window and fragmented by higher energy collisional dissociation (HCD) with normalized collision energy of 35%, then measured in the linear ion trap using the rapid ion trap scan rate. Automatic gain control targets were 5 × 10e5 ions with a max injection time of 50 ms for full scans and 5 × 10e3 with 35 ms for MS/MS scans. Dynamic exclusion time was set as 18 s.

The MS analysis for Q Exactive HF were performed with one full scan (300–1400 m/z, *R* = 60,000 at 200 m/z) at automatic gain control target of 3e6 ions, followed by up to 20 data-dependent MS/MS scans with HCD (target 2 × 10e3 ions, max injection time 40 ms, isolation window 1.6 m/z, normalized collision energy of 27%), detected in the Orbitrap (*R* = 15,000 at 200 m/z).

### MS data processing

All the MS data were processed in the Firmiana^60^ platform. Raw files were searched against the human National Center for Biotechnology Information (NCBI) ref-seq protein database (updated on 07-04-2013, 32,015 entries) by Mascot 2.3 (Matrix Science Inc). Mass tolerances were 20 ppm for precursor and 0.5 Da for products ions for Fusion and Fusion Lumos series. Mass tolerances were 20 ppm for precursor and 50mmu for products ions for Q Exactive HF series. Up to two missed cleavages were allowed. The data were also searched against a decoy database so that protein identifications were accepted at a false discovery rate (FDR) of 1%.

For proteome profiling, Carbamidomethylation (C) was set in search engine as a fixed modification; Acetyl (Protein N-term) and Oxidation (M), as variable modifications. For phospho-proteome, Carbamidomethylation (C) was set in search engine as a fixed modification; Phospho (ST), Phospho (Y), Acetyl (Protein N-term), and Oxidation (M), as variable modifications. Phospho-sites were reported when phospho-peptides showed an ion score >20, otherwise the precise modification site was deemed ambiguous. Phospho-sites with abundance <25% of all phospho-sites were excluded. For TF activity profiles, the search engine set Phospho (ST), Phospho (Y), DeStreak (C), Acetyl (Protein N-term), and Oxidation (M) as variable modifications.

### Protein quantification and normalization

We applied match between runs (MBR) algorithm^60,61^. We built a dynamic regression function based on common identified peptides in samples. According to correlation value R^2^, Firmiana chose linear or quadratic function for regression to calculate retention time (RT) of corresponding hidden peptides, and to check the existence of the extracted ion chromatogram (XIC) based on the m/z and calculated RT. The function evaluated the peak area values of those existed XICs. These peak area values were considered as parts of corresponding proteins.

For proteomic data normalization, label-free protein quantifications were calculated using a label-free, intensity based absolute quantification (iBAQ) approach^62^. The fraction of total (FOT) was used to represent the normalized abundance of a particular protein across samples. FOT of protein was defined as a protein’s iBAQ divided by the total iBAQ of all identified proteins within one sample. The FOT was multiplied by 10e6 for the ease of presentation. For the phospho-proteomics, the data matrix of peptides with phosphorylated modification was used for phospho-sites extraction and quantification. Then, the phospho-sites expression matrix was subjected to quantile normalization using normalized quantile functions^22,63^ implemented in the R/Bioconductor package limma v.3.24.15^64^. After that, the normalized phospho-sites abundance was log2-transformed. We obtained a quantified data matrix including 44,750 phospho-sites (Supplementary Data 2c). In TF activity profile, we also used quantile-based normalization and obtained a quantified data matrix including 597 TFs (Supplementary Data 2b). The data distribution (Supplementary Fig. 1c) showed quantile normalization was suitable for our TF activity profile, too. At last, missing values were assigned the minimum value in each proteomic layer.

### Quality control (QC) for MS platforms and samples data

QC was performed for platforms and samples. The average spearman’s correlation coefficient among standards (tryptic digestions of the HEK293T cell lysate, Cat# CRL-11268 from ATCC; RRID: CVCL_QW54) in proteome platform was 0.92; the average correlation coefficient among standards in TF activity profile platform was 0.95; and the average correlation coefficient among standards in phospho-proteome platform was 0.94 (Supplementary Fig. 1a). The median cv values among standards in proteome, phospho-proteome, and TF activity profile platforms were 0.28, 0.26, and 0.34, respectively (Supplementary Fig. 1b). The density of the tumor (orange) and NAT (blue) proteomes exhibited a unimodal distribution, in accordance with the proteomic quality control (Supplementary Fig. 1c). These results showed the stability of our MS platforms.

For samples data, the distribution of median values was used to discriminate the samples with insufficient protein or phospho-site detected. The samples with median values which were larger than upper quartile + 1.5 IQR (interquartile range) would be excluded from further analyses. To evaluate the comparability of data, we compared the data distribution with boxplots and density curves. Samples with a clear bimodal distribution of protein quantification would be excluded from further analyses. Furthermore, QC results required both of tumor tissues and paired NATs passed QC procedures. In this research, 194 pairwise samples of proteomic profiles, 196 pairwise samples of TF activity profiles, and 184 pairwise samples of phospho-proteomic profiles passed the QC procedures and were used for further analyses.

### Principal component analysis (PCA)

PCA was performed to visualize the separation of tumor tissues and normal adjacent tissues (NATs). We performed PCA on 196 paired tumor and NAT samples to illustrate the proteomic, phospho-proteomic, and TF activity profile differences between tumor and NAT samples (Supplementary Fig. 2a). Also, we performed PCA on 196 DGC, IGC, and MGC samples’ TF activity profiles to illustrate the global molecular differences between Lauren classification of GC samples (Supplementary Fig. 5d). The PCA function under the R package was implemented for unsupervised clustering analysis. The 90% confidence coverage was represented by a colored ellipse for each group, which was calculated based on the mean and covariance of points in each specific group.

### The screen of differently expressed proteins (DEPs)

Wilcoxon paired signed-rank test was used to identify proteins with significantly differential expression between tumor tissues and NATs. Wilcoxon rank-sum test was used to identify proteins with significantly differential expression between DGC and IGC. DEPs were also examined between two clusters of TF activity-based subtypes by Wilcoxon rank-sum test in DGC and IGC, respectively. *P*-values were adjusted using Benjamini-Hochberg (BH) correction. Foldchange was calculated by average or median ratio. Proteins with foldchange values larger than certain standards (usually 2x) and BH adjusted *p*-values < 0.05 were considered as significantly different.

### Pathway enrichment analysis

DEPs or subtype signature proteins were used to perform pathway enrichment analysis according to Gene Ontology and KEGG in DAVID. Reactome or STRING-based pathway enrichment analysis was also performed. Statistical significance was considered when FDR value was <0.05.

### Kinase-substrate enrichment analysis (KSEA)

Kinase-Substrate Enrichment Analysis (KSEA) estimated changes in a kinase’s activity by measuring and averaging the amounts of its identified substrates instead of a single substrate, which enhanced the signal-to-noise ratio from inherently noisy phospho-proteomic data. The ratios of identified phospho-sites between tumor tissues and NATs were used to estimate the kinase activities by KSEA algorithm^28^. The information of kinase-substrate relationships was obtained from databases including PhosphoSite^65^ and NetworKIN 3.0. Statistical analysis was performed in R (version 4.0.4) with Kruskal–Wallis test.

### TFRE enrichment analysis^66^

We calculated the proteins ratio between proteome and TF activity profiles. The TFs, which were annotated in CellNet^38^ database, had higher ratio than other proteins. Besides TFs, we selected proteins with ratio values >4 folds as TFRE enriched proteins. In total, 4185 proteins were regarded as TFRE enriched proteins, including 597 TFs.

### Gene set enrichment analysis (GSEA)

Gene Set Enrichment Analysis (GSEA) was applied to find enriched pathways between tumor tissues and NATs. Proteins detected in >95% samples were selected, and missing values were then imputed with the minimum value of the proteomic data. It was also used to calculate the GSEA enrichment scores over 4347 pathways with at least 10 overlapping genes, for each sample. GSEA was performed by the GSEA software (http://software.broadinstitute.org/gsea/index.jsp) or R package clusterProfiler. Gene sets including Gene ontology, KEGG, Reactome, and HALLMARK downloaded from the Molecular Signatures Database (MSigDB v7.1, http://software.broadinstitute.org/gsea/msigdb/index.jsp) were set as background.

### Tissue specific proteins analysis

Tissue specific annotation was from Human Protein Atlas^67^. In total, 1882 proteins had tissue-specific annotation in proteomic data, including 206 TFs. We calculated the proportion of tissue-specific proteins alteration in each tissue, especially digestive tract including esophagus, intestine, liver and stomach. TF-TG regulated network was built based on gene regulatory network from CellNet^38^.

### Kaplan–Meier analysis

Standard statistical tests were used to analyze the clinical data, including but not limited to student’s *t*-test, Fisher’s exact test, and Log-rank test. All survival analysis among the proteomic/TF activity/immune subtypes, used Kaplan–Meier method; *p*-values were calculated using the Log-rank test. Hazard ratio (HR) was calculated from Cox proportional hazards regression analysis. All the survival analyses of proteomic subtyping were adjusted by other clinical covariates including gender, age, TNM stage and chemotherapy, demonstrating that our subtyping could serve as an independent survival outcome predictive factor. In addition, we performed formal statistical tests for interaction analyses. The results of interaction analyses revealed there was no significant enrichment of TNM stages in each subtype. All subtyping survival outcome analyses results were shown by DFS. For the optimal cutoff point in the K-M analysis of certain proteins, we used function *surv_cutpoint* of *survminer* package in R. *P*-values < 0.05 were considered as significantly different. All the analyses of clinical data were performed in R or GraphPad Prism.

### Consensus clustering analysis

Consensus clustering was performed using the R package *Consensus Cluster Plus*. Samples were clustered using Euclidean distance as the distance measure. We performed 1000 resampling repetitions in the range of 2 to 6 clusters. Log-rank tests and Kaplan–Meier survival curves were used to compare the survival curves among the subtypes.

The protein expression matrix of the 79 paired DGC samples was used to identify the DGC proteomic subtypes with upregulated proteins in tumor tissues. The protein expression matrix of the 92 paired IGC samples was used to identify the IGC proteomic subtypes with upregulated proteins in tumor tissues. As summarized in Supplementary Fig. 6a, the clustering analysis of the tumors by protein abundance divided DGC and IGC patients into three proteomic subtypes, respectively. A consensus matrix with *k* = 3 appeared to have the clearest cut between clusters and showed significant association with the patients’ survival. Thus, we selected 3 clusters as the best subtypes for the DGC and IGC proteomic subtypes.

For the TF activity profiles, 425 and 396 TFs detected in >50% DGC and IGC patients, respectively, were applied for DGC and IGC subtyping. We performed consensus clustering and set the same parameters as that for the proteomic subtyping. The consensus CDF and delta plots showed increasing in area for *k* = 2, and this provided the clearest separation among the clusters (Supplementary Fig. 8a, b). Thus, we selected 2 clusters as the best subtypes for the TF activities matrix.

For the phospho-proteome data, the phospho-sites detected in >50% DGC and IGC patients, corresponding to 4484 and 4739 phospho-proteins, respectively, were applied for DGC subtyping and IGC subtyping. We performed consensus clustering and set the same parameters as that for the proteomic subtyping. The consensus cumulative distribution function (CDF) and delta plots showed increasing in area for *k* = 3, and this provided the clearest separation among the clusters (Supplementary Fig. 9a). Thus, we selected 3 clusters as the best subtypes for the phospho-proteomic expression matrix.

Consensus clustering was performed with the xCell results of 183 paired GC samples.

Euclidean distance and 1000 resampling repetitions in the range of 2–6 clusters were used. As summarized in Supplementary Fig. 11a, the clustering analysis of the tumors by xCell score divided 183 patients into three immune clusters. A consensus matrix with *k* = 3 appeared to have the clearest cut between clusters and showed significant association with the patients’ survival in DGC and IGC. Thus, we selected 3 clusters as the best subtypes for the inferred immune cell score matrix.

To identify molecular signatures for each subtype in our proteomic cohort, we compared the protein expression in each subtype against all other subtypes. The statistical significance was calculated by Wilcoxon rank-sum test. For a given subtype, proteins with a foldchange > 2 and *p* < 0.05, were defined as signature proteins, when compared with other subtypes.

### Bayesian predictor for NFKB1 and SMARCC1 subtypes

The Bayesian algorithm was applied to cluster subtypes based on TF activity in Mun’s cohort (Fig. 6h, NFKB1 subtype and SMARCC1 subtype)^47^. The z scores of NFKB1 and SMARCC1 in our TF activity profiles were used to create a linear predictor score (LPS) for each patient based on TF activity subtypes. The LPS distribution of each TF activity subtype was used to estimate the likelihood that a new sample was in each of the two subtypes by applying Bayes’ rule. The z scores of NFKB1 and SMARCC1 in the validation cohort were used to calculate the probability based on the predictor. The membership of NFKB1 and SMARCC1 subtype was assigned as above based on a cutoff of 75% certainty. At last, 28 and 24 cases were identified as NFKB1 subgroup and SMARCC1 subgroup, respectively.

### Classification concordance evaluation

Based on the classification among proteomic subtypes, phospho-proteomic subtypes and TF activity-based subtypes of our cohort, we performed chi-square test to assess the classification concordance. Except correspondence between phospho-proteomic subtypes and TF activity-based subtypes in IGC, the statistical results of classification concordance among subtypes based on three datasets were all significant (chi-square test, *p* < 0.05, Supplementary Table 2). Based on the classification of Mun’s cohort^10^, we performed chi-square test to assess the classification concordance among proteomic subtypes, TF subtypes, and Mun’s subtypes. The statistical results of classification concordance among subtypes were significant (chi-square test, *p* < 0.05, Supplementary Fig. 10f). These results demonstrated that our subtypes had high classification concordance.

### Cell cycle phase analysis

Cell cycle phase analysis was performed using the R package *Seurat*^68^. Cell cycle scores of patients were calculated and patients were labeled with G1, S and G2/M classification (Fig. 4f). Significantly upregulated cell cycle regulating proteins and phospho-sites were selected (foldchange > 2, Wilcoxon rank-sum test, BH adjusted *p* < 0.05).

### Master TFs nomination

Master TFs dominate the GC progression. We nominated master TFs according to three criteria^66^ as follows: (a) the activities of TFs were upregulated in tumor tissues comparing to NATs; (b) the activities of TFs were upregulated in a subtype; (c) significant enrichment based on altered TGs. In Supplementary Fig. 8e, enrichment was calculated based on DEPs between DGC and IGC using hypergeometric test. In Fig. 5c, enrichment was calculated based on DEPs between two TF activity-based subtypes. *P*-values < 0.05 were considered as significant enrichment.

### Construction of signaling transduction network

In Fig. 5h, i, the network among kinases and TFs were annotated using calculated correlation between TFs’ phospho-sites and kinases. Four phospho-sites were considered as key phospho-sites which affected the TFs’ activities. The correlation between the kinase activities and the phospho-sites on TFs were calculated with pair-wised spearman’s correlation coefficients. *P*-values < 0.05 were considered as significant correlation. Forty significantly positive correlations were used to construct the kinase-TF regulation network (Fig. 5h-i). TGs were from DEPs between two TF activity-based subtypes. The TF-TG regulated network was built on CellNet^38^, visualized and generated by the software Cytoscape (version 3.6.1).

### xCell

The abundance of 64 kinds of cell types, microenvironment scores, immune scores, and stroma scores were inferred by proteome data via xCell (https://xcell.ucsf.edu/). The density distribution was generated based on immune and stroma scores. The differential cell types between DGC and IGC in immune cluster 3 were compared based on xCell scores.

### Reporting summary

Further information on research design is available in the Nature Portfolio Reporting Summary linked to this article.

## Supplementary information

Supplementary Information

Description of Additional Supplementary Files

Supplementary Data 1

Supplementary Data 2

Supplementary Data 3

Supplementary Data 4

Supplementary Data 5

Supplementary Data 6

Supplementary Data 7

Reporting Summary

## Data Availability

The MS raw data generated in this study have been deposited in the ProteomeXchange Consortium (dataset identifier: PXD038214) via the iProX^69^ partner repository under accession code IPX0004428000. The normalized proteome, phospho-proteome, and TF activity data matrices are available under this accession. The MS raw data of anti-PD1 group have been deposited in the ProteomeXchange Consortium (dataset identifier: PXD038188) via the iProX^69^ partner repository under accession code IPX0004819000. The targeted exome sequencing data are available in the GSA^70^ (Genome Sequence Archive, https://ngdc.cncb.ac.cn/gsa-human/) under restricted access HRA002466 and HRA003612 (fastq files) for data privacy laws related to patient consent for data sharing, access can be obtained by the Request Data steps in GSA database website or contacting corresponding author. The approximate response time for accession requests is about 2 weeks. Once access has been granted, the data will be available to download for 3 months. The TCGA publicly available data used in this study are available in the Genomic Data Commons Data Portal under accession code TCGA-STAD (https://portal.gdc.cancer.gov/)^7^. The remaining data are available within the Article, Supplementary Information or Source Data file. Source data are provided with this paper.

## Source data

Source Data
