# Supplementary material for: Multilevel proteomic analyses reveal molecular diversity between diffuse-type and intestinal-type gastric cancer
Source: Nat Commun. 2023 Feb 14;14:835. doi: 10.1038/s41467-023-35797-6 (PMC9929250; doi:10.1038/s41467-023-35797-6)
Supplement: Supplementary file 1 — Supplementary Information [file 41467_2023_35797_MOESM1_ESM.pdf]

Characteristic	DGC (n=83)	IGC (n=102)
**Age ------no.(%)**		
≥50	62 (75%)	89 (87%)
<50	21 (25%)	13 (13%)
**Gender ------no.(%)**		
Male	55 (66%)	79 (77%)
Female	28 (34%)	23 (23%)
**TNM stage ------no.(%)**		
I	5 (6%)	15 (15%)
II	24 (29%)	33 (32%)
III	50 (60%)	50 (49%)
IV	4 (5%)	4 (4%)
**Chemotherapy ------ no.(%)**		
Chemotherapy	65 (78%)	73 (72%)
No Chemotherapy	18 (22%)	29 (28%)
**Lymphovascular invasion------no.(%)**		
Positive	53 (64%)	38 (37%)
Negative	30 (36%)	62 (61%)
Unknown		2 (2%)
**Live status------no.(%)**		
Dead	35 (42%)	35 (34%)
Alive	48 (58%)	67 (66%)
**Disease free survival------no.(%)**		
Yes	33 (40%)	38 (37%)
No	46 (55%)	63 (62%)
Unknown	4 (5%)	1 (1%)

Supplementary Table 1. The baseline clinical characteristics of GC patients. Patient numbers are shown in table.

Subtypes	Patient numbers		
**DGC**			
Proteomic subtypes	D-1	D-2	D-3
TF activity subtypes D-1	15	18	3
TF activity subtypes D-2	8	10	25
p value			0.0001
Phosphoproteomic subtypes	D-1	D-2	D-3
TF activity subtypes D-1	20	14	3
TF activity subtypes D-2	7	23	13
p value			0.0008
Proteomic subtypes	D-1	D-2	D-3
Phosphoproteomic subtypes D-1	10	11	2
Phosphoproteomic subtypes D-2	9	13	15
Phosphoproteomic subtypes D-3	2	3	11
p value			0.003
**IGC**			
Proteomic subtypes	I-1	I-2	I-3
TF activity subtypes I-1	11	23	5
TF activity subtypes I-2	7	25	20
p value			0.0159
Phosphoproteomic subtypes	I-1	I-2	I-3
TF activity subtypes I-1	12	11	7
TF activity subtypes I-2	15	14	22
p value			0.1668
Proteomic subtypes	I-1	I-2	I-3
Phosphoproteomic subtypes I-1	2	24	1
Phosphoproteomic subtypes I-2	22	1	7
Phosphoproteomic subtypes I-3	2	11	14
p value			5.36E-13

Supplementary Table2. The statistical analysis of the correspondence among subtypes from three datasets. Patient numbers are shown in table. P values by two-sided chi-square test.

Supplementary Figure 1

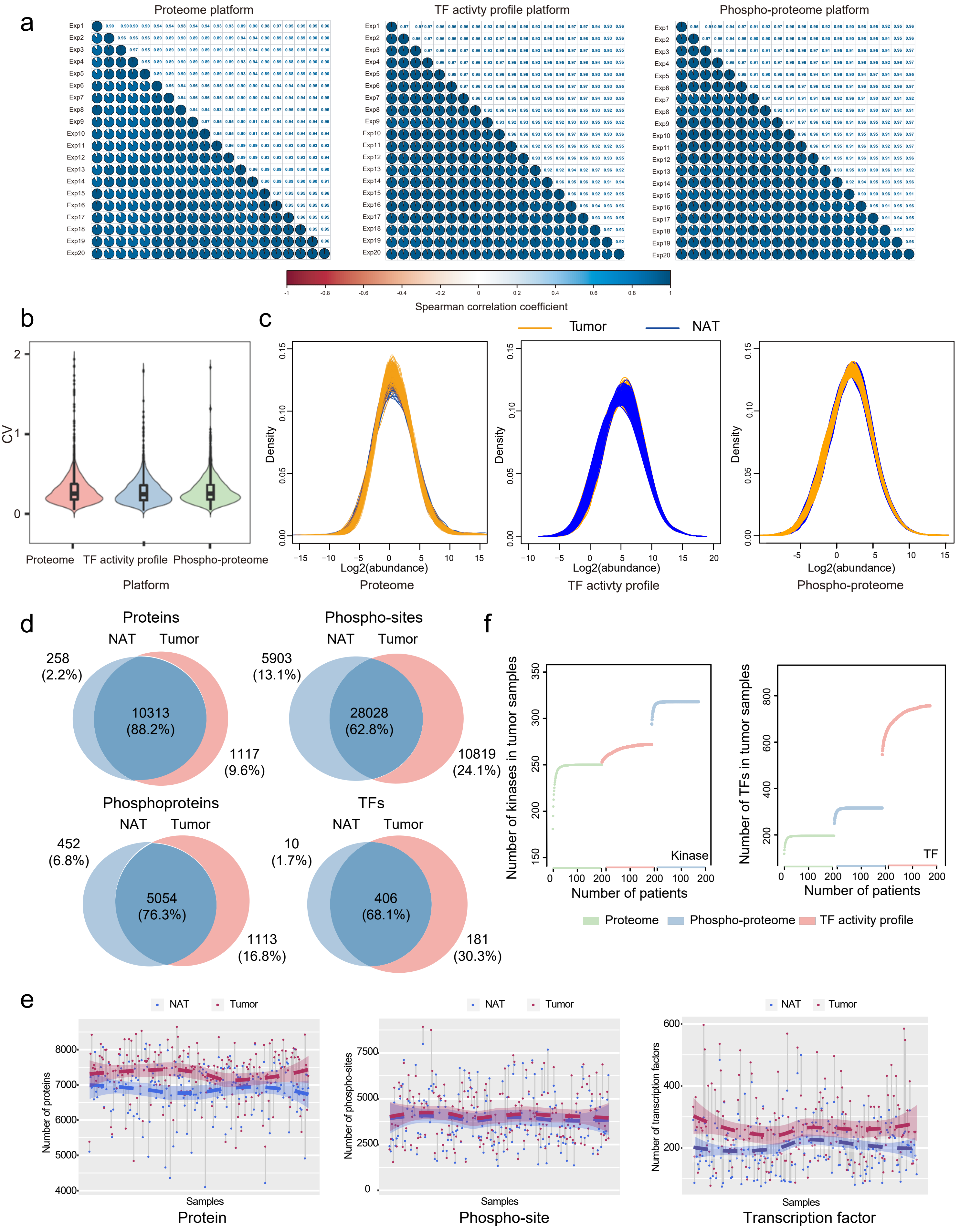

Supplementary Figure 1. **Construction of human GC multilevel proteomic atlas.** Related to Figure 1. **a**, Correlation analysis of twenty 293T samples as MS quality control to evaluate the robustness of label-free quantification. Top-right half panel: pairwise calculation of spearman's correlation coefficients between twenty samples; bottom-left half panel: pairwise comparison of twenty samples by scatterplots. **b**, The CV distribution of three platforms. n(standards)=20 in each platform. Violin plots showed median and interquartile range. **c**, Density plots indicating distribution of protein abundance in tumor tissues (orange) and NATs (blue). **d**, Overview of the proteome, phospho-proteome and TF activity profiles of GC patients. Shown are the numbers of proteins, phospho-sites, phosphoproteins and TFs identified in tumor tissues and NATs. **e**, Pairwise comparison of proteins, phospho-sites, and TFs identified in tumor tissues (red dots) and NATs (blue dots). n (proteome) =194, n (phospho-proteome) = 184, and n (TF activity profile) = 196 biologically independent samples. The dashed curves fitted by lasso regression showed the distribution of protein identifications. The shading that underlies the lasso curves denoted the 95% confidence intervals. **f**, Saturation curve of kinases and TFs identification. Different colors indicate different datasets.

Supplementary Figure 2

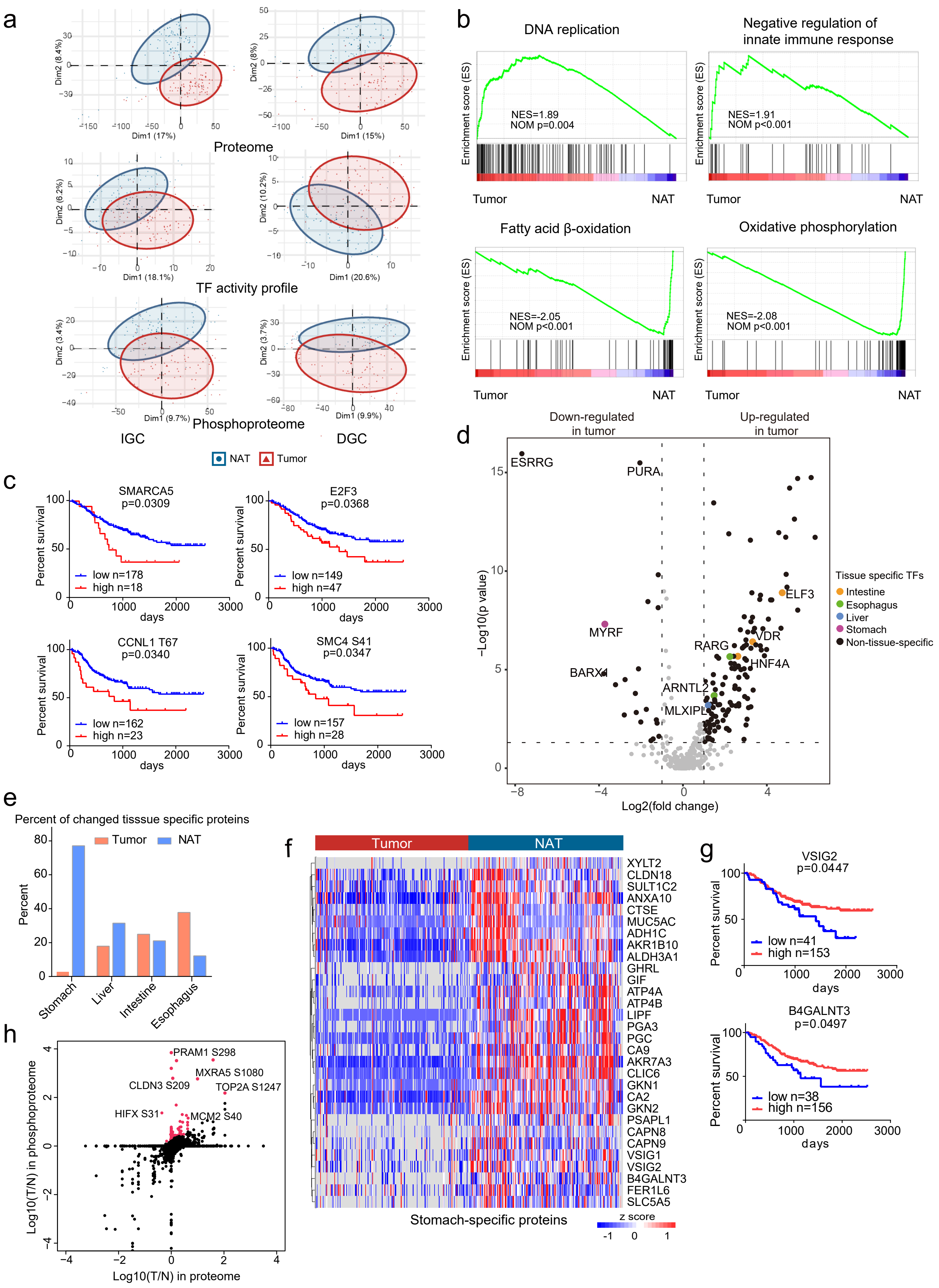

Supplementary Figure 2. **Multilevel proteomic features of tumor tissues and NATs in GC.** Related to Figure 1. **a**, PCA of proteome, phospho-proteome, and TF activity profiles in 196 paired GC patients. Red, tumor tissues; blue, NATs. Left, IGC; right, DGC. **b**, GSEA revealed overrepresented pathways in tumor tissues and NATs. **c**, Clinical outcomes of TFs and phospho-sites involved in cell cycle. SMARCA5: n (low) = 178 and n (high) = 18 biologically independent samples. E2F3: n (low) = 149 and n (high) = 47 biologically independent samples. CCNL1 T67: n (low) = 162 and n (high) = 23 biologically independent samples. SMC4 S41: n (low) = 157 and n (high) = 28 biologically independent samples. P values were from Log-rank test. **d**, A volcano plot showing the differential TFs activity between tumor tissues and NATs. Tissue specific TFs are shown. The p values were from Wilcoxon paired signed-rank test, and adjusted using Benjamini-Hochberg (BH) correction. **e**, Percentage of upregulated tissue specific proteins in tumor tissues and NATs. **f**, A list of gastric specific proteins that were differentially expressed in tumor tissues and NATs. **g**, VSIG2 and B4GALNT3 were significantly associated with prognoses (p values were from Log-rank test). VSIG2: n (low) = 41 and n (high) = 153 biologically independent samples. B4GALNT3: n (low) = 38 and n (high) = 156 biologically independent samples. **h**, Foldchanges of phospho-sites and corresponding proteins in tumor tissues and NATs. Red dots (defined as cancer related phosphoproteins): changes of phosphoproteins abundance were greater than changes of their corresponding proteins abundance. Source data are provided as a Source Data file.

Supplementary Figure 3

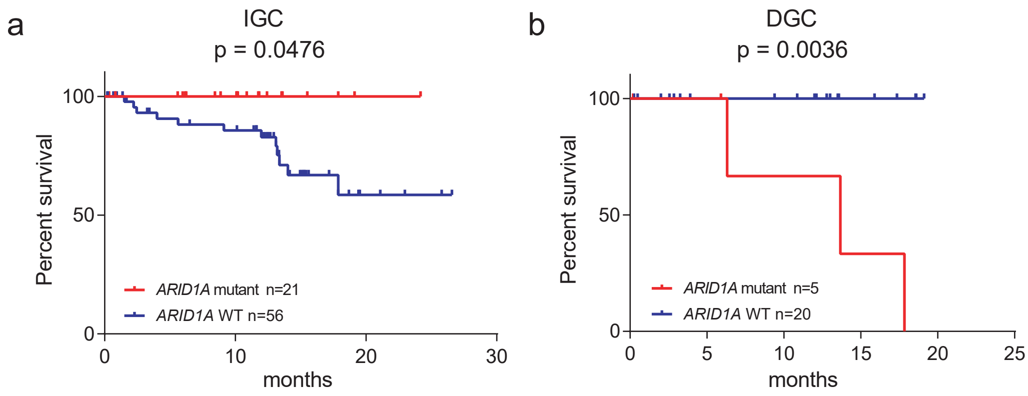

Supplementary Figure 3. The clinical outcomes of *ARID1A* mutant patients in TCGA cohort. Related to Figure 2. Log-rank test was performed. **a**, The clinical outcomes of *ARID1A* mutant patients in IGC. n (mutant) = 21 and n (WT) = 56 biologically independent samples. **b**, The clinical outcomes of *ARID1A* mutant patients in DGC. n (mutant) = 5 and n (WT) = 20 biologically independent samples.

Supplementary Figure 4

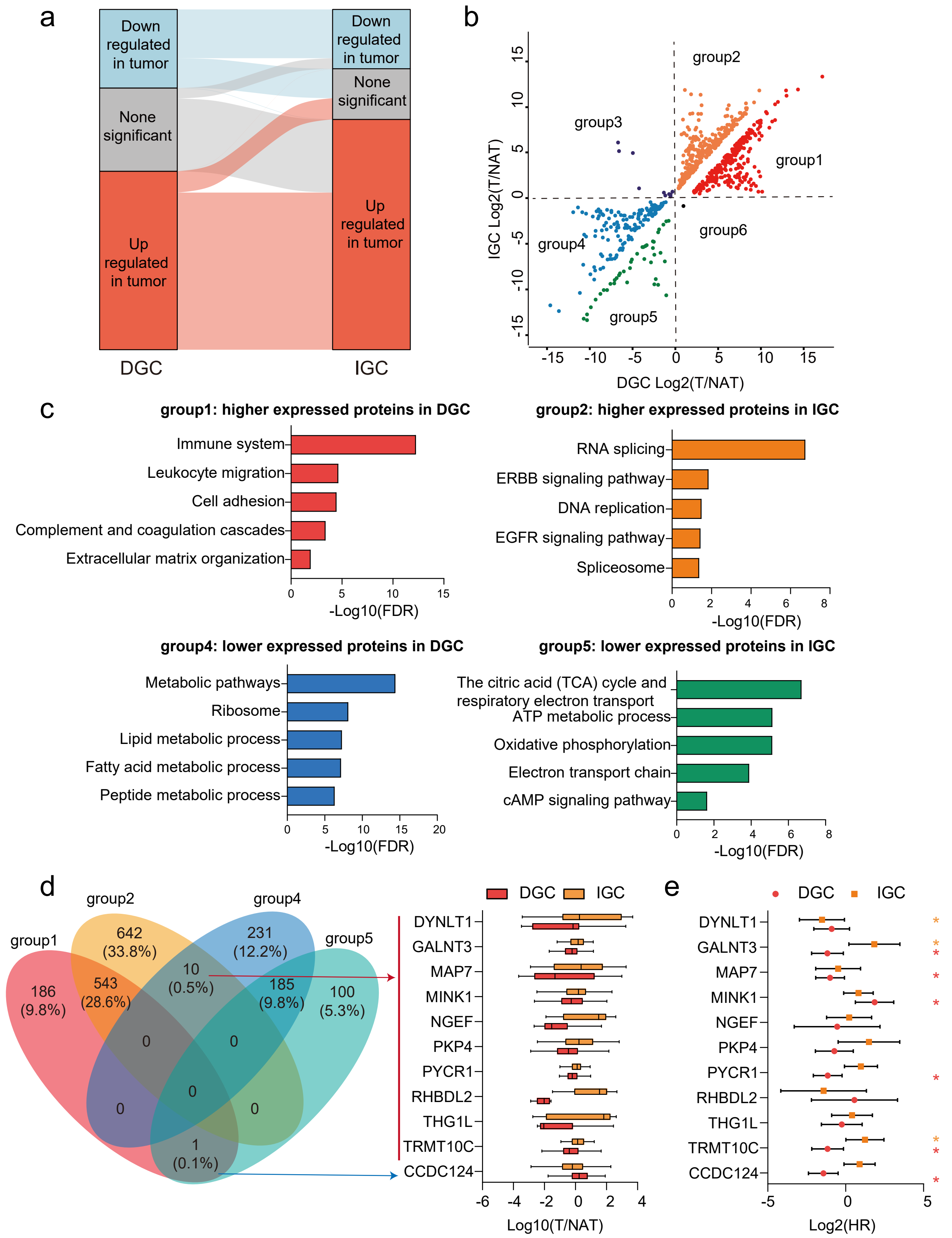

Supplementary Figure 4. The direction of dysregulated proteins in DGC and IGC. Related to Figure 3. **a**, The correspondence of up-regulated proteins in tumor tissues (red) and down-regulated proteins in tumor tissues (blue) between DGC and IGC. **b**, Six groups of dysregulated proteins. **c**, Pathway enrichment analysis with differently expressed proteins in four main groups. **d**, The overlap of dysregulated proteins in four groups. n (DGC) = 79 and n (IGC) = 92 biologically independent samples. Boxplots showed median (central line), upper and lower quartiles (box limits), min to max range. **e**, The relationship between proteins and prognoses in DGC and IGC. The significant correlation was marked with the asterisk. n (DGC) = 79 and n (IGC) = 92 biologically independent samples. The points and error bars showed the median of hazard ratio (HR) and 95% confidence interval (CI). Source data are provided as a Source Data file.

Supplementary Figure 5

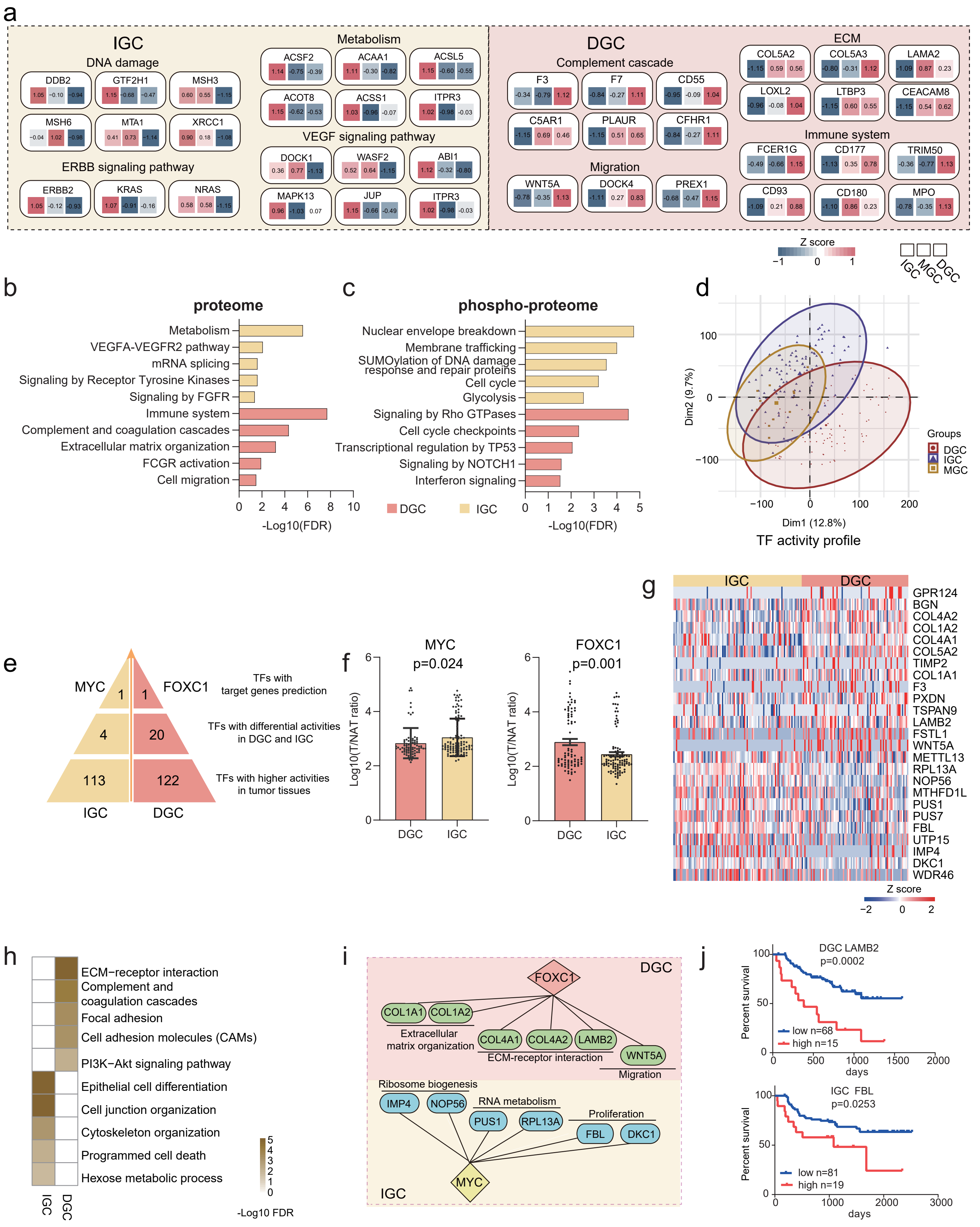

Supplementary Figure 5. Integrated analysis of differentially activated pathways at protein, TF activity and phosphoprotein levels in DGC and IGC. Related to Figure 3. **a**, Representative differentially expressed proteins in overrepresented pathways of IGC and DGC. **b**, Pathway analysis of differentially expressed proteins between tumor tissues and NATs in IGC and DGC. **c**, Pathway analysis of differentially expressed phosphoproteins between IGC and DGC. **d**, PCA of TF activity profiles in 196 GC patients. Red, DGC; blue, IGC; orange, MGC. **e**, Identification of master TFs maintaining pathological types. **f**, The activities of master TFs in DGC and IGC. n (DGC) = 83 and n (IGC) = 102 biologically independent samples. The data was shown as mean ± SEM. P-values were calculated using two-sided student's t test. **g**, The expression of TGs regulated by master TFs in DGC and IGC. **h**, Pathway analysis of TGs regulated by altered TFs in DGC and IGC. **i**, The master TF and TG pairs in DGC and IGC. **j**, The association of TGs with clinical outcomes. LAMB2: n (low) = 68 and n (high) = 15 biologically independent samples. FBL: n (low) = 81 and n (high) = 19 biologically independent samples. P values were from Log-rank test. Source data are provided as a Source Data file.

Supplementary Figure 6

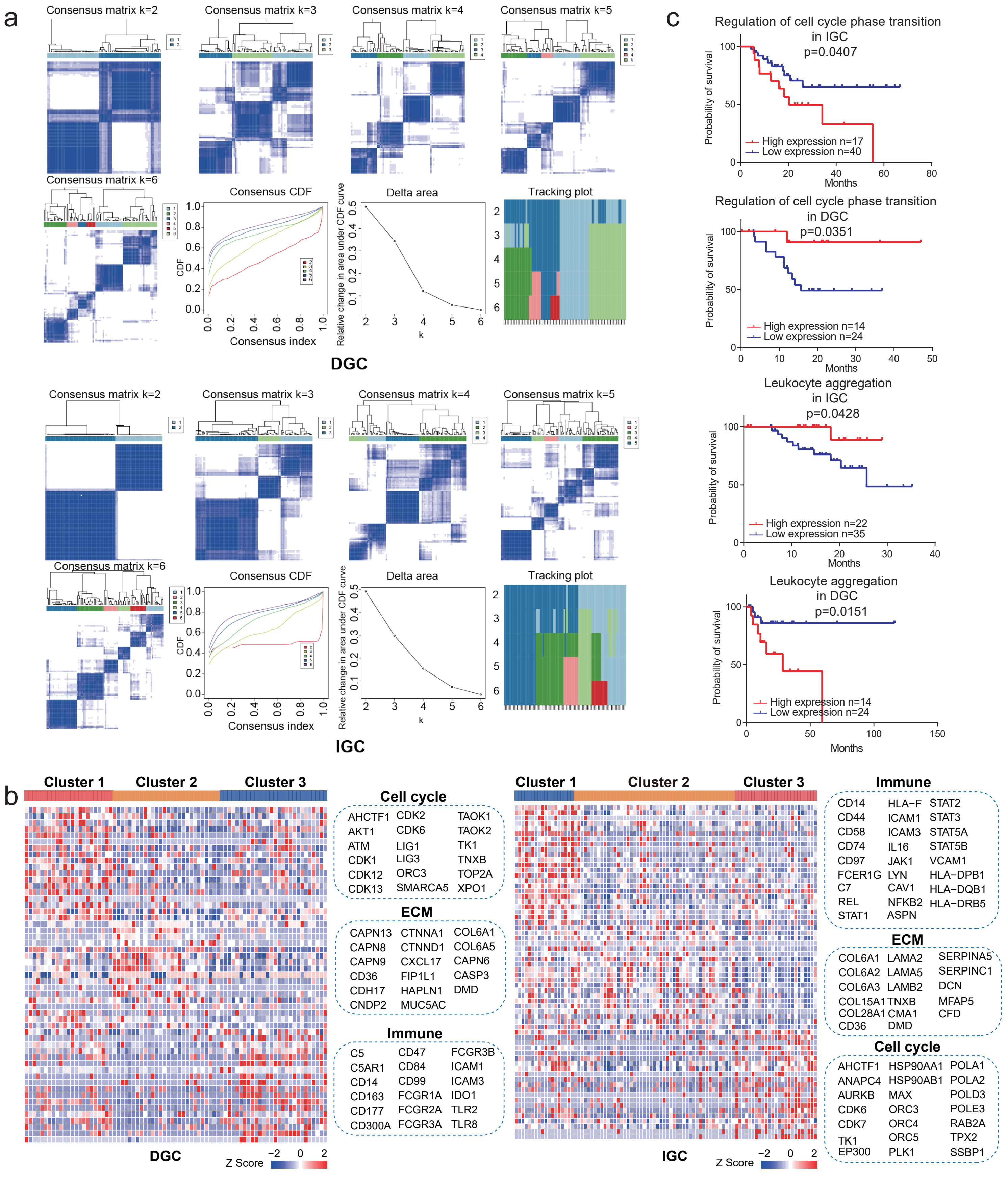

Supplementary Figure 6. **Proteomic subtypes of GC patients.** Related to Figure 4. **a**, Consensus matrices of identified clusters (k = 2 to 6) of DGC and IGC proteomic subtypes. **b**, Representative differentially expressed proteins in the overrepresented pathways among three DGC and IGC proteomic subtypes. **c**, ssGSEA revealed the cell cycle and immune related pathways had opposite prognoses between DGC and IGC. Regulation of cell cycle phase transition in IGC: n (low) = 40 and n (high) = 17 biologically independent samples. Regulation of cell cycle phase transition in DGC: n (low) = 24 and n (high) = 14 biologically independent samples. Leukocyte aggregation in IGC: n (low) = 35 and n (high) = 22 biologically independent samples. Leukocyte aggregation in DGC: n (low) = 24 and n (high) = 14 biologically independent samples. The p values were from Log rank test.

Supplementary Figure 7

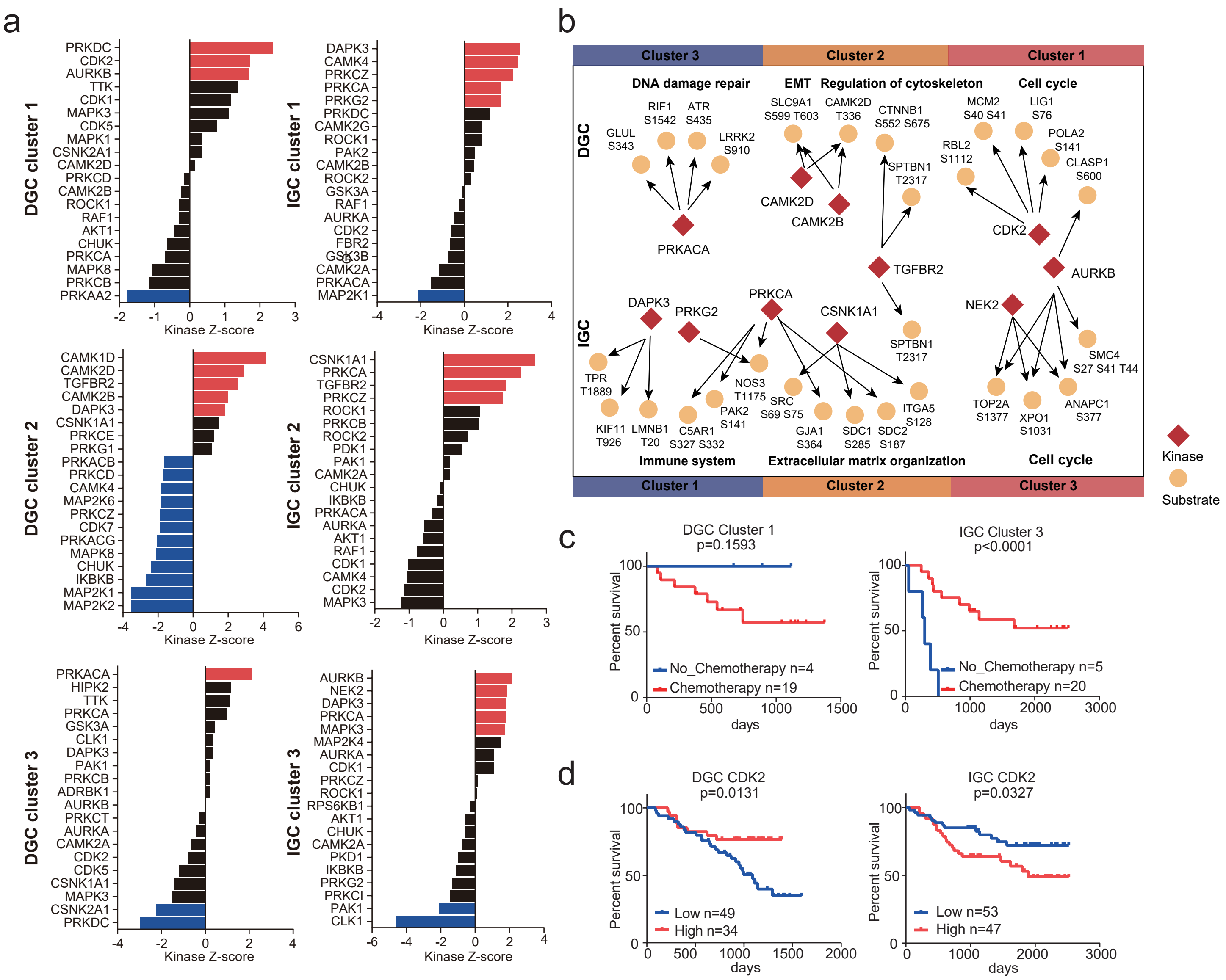

Supplementary Figure 7. **Kinases alteration in proteomic subtypes.** Related to Figure 4. **a**, KSEA analysis of kinase activities in each GC proteomic subtype. **b**, Summary of the kinases and their corresponding phospho-sites and the activated pathways in the proteomic subtypes. **c**, The association of adjuvant chemotherapy with clinical outcomes in DGC cluster 1 and IGC cluster 3. DGC cluster 1: n (chemotherapy) = 19 and n (no chemotherapy) = 4 biologically independent samples. IGC cluster 3: n (chemotherapy) = 20 and n (no chemotherapy) = 5 biologically independent samples. P values were from Log-rank test. **d**, The association of CDK2 activity with clinical outcomes in DGC and IGC. DGC: n (low) = 49 and n (high) = 34 biologically independent samples. IGC: n (low) = 53 and n (high) = 47 biologically independent samples. P values were from Log-rank test. Source data are provided as a Source Data file.

Supplementary Figure 8

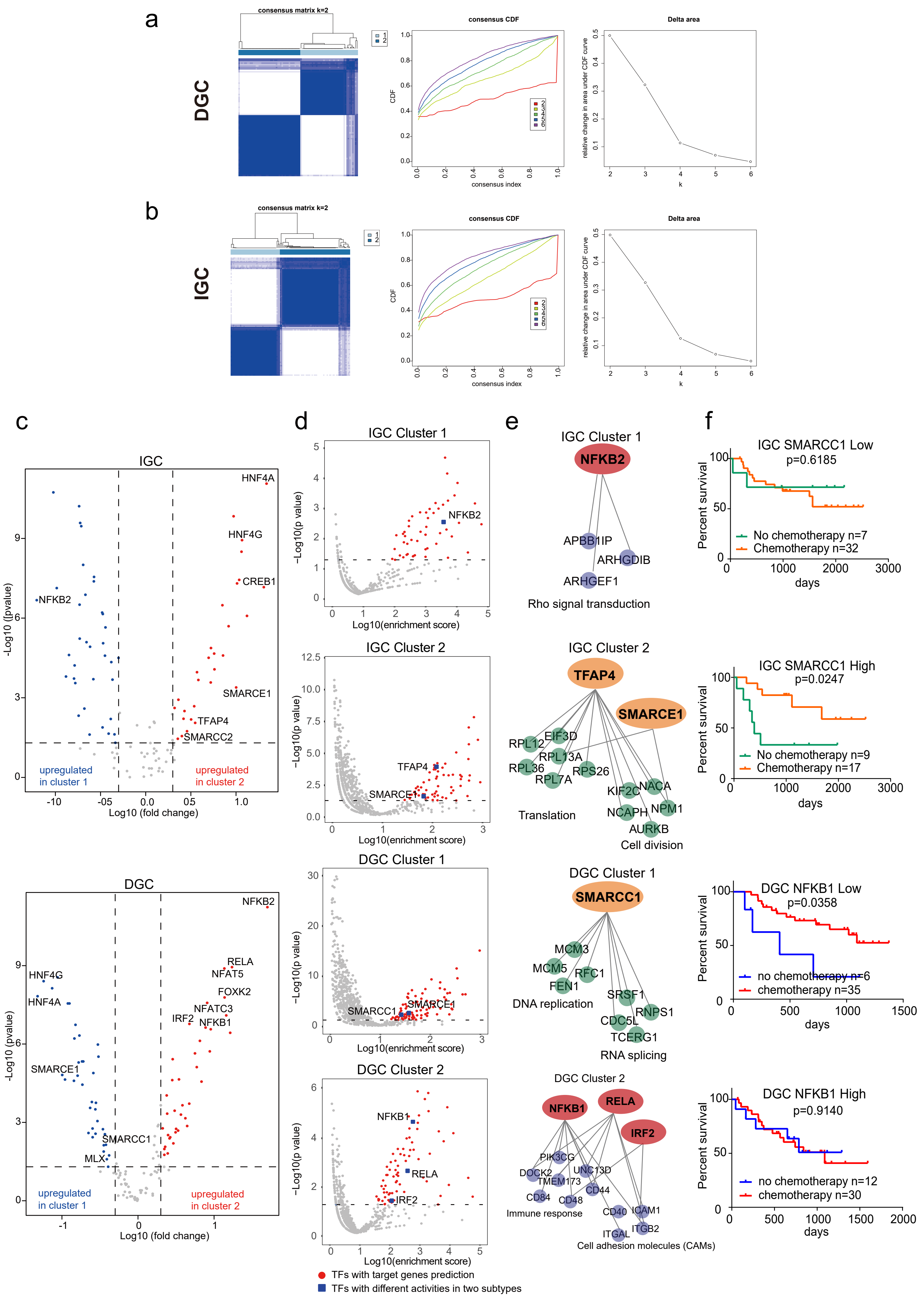

Supplementary Figure 8. DGC and IGC subtypes based on TF activity profiles. Related to Figure 5. **a**, Consensus clustering analysis of TF activity profiles identified two subtypes in DGC. **b**, Consensus clustering analysis of TF activity profiles identified two subtypes in IGC. **c**, Volcano plots depicted the differential abundance of signature TFs in TF activity-based subtypes. Wilcoxon rank-sum test with Benjamini-Hochberg (BH) adjustment. **d**, Master TFs selection based on protein expression profiling. Hypergeometric test. **e**, TF-TG regulation network in TF activity-based subtypes of DGC and IGC. **f**, The association of adjuvant chemotherapy with DFS in patient groups with or without high master TF activities. IGC SMARCC1 low: n (chemotherapy) = 32 and n (no chemotherapy) = 7 biologically independent samples. IGC SMARCC1 high: n (chemotherapy) = 17 and n (no chemotherapy) = 9 biologically independent samples. DGC NFKB1 low: n (chemotherapy) = 35 and n (no chemotherapy) = 6 biologically independent samples. DGC NFKB1 high: n (chemotherapy) = 30 and n (no chemotherapy) = 12 biologically independent samples. P values were from Log-rank test.

Supplementary Figure 9

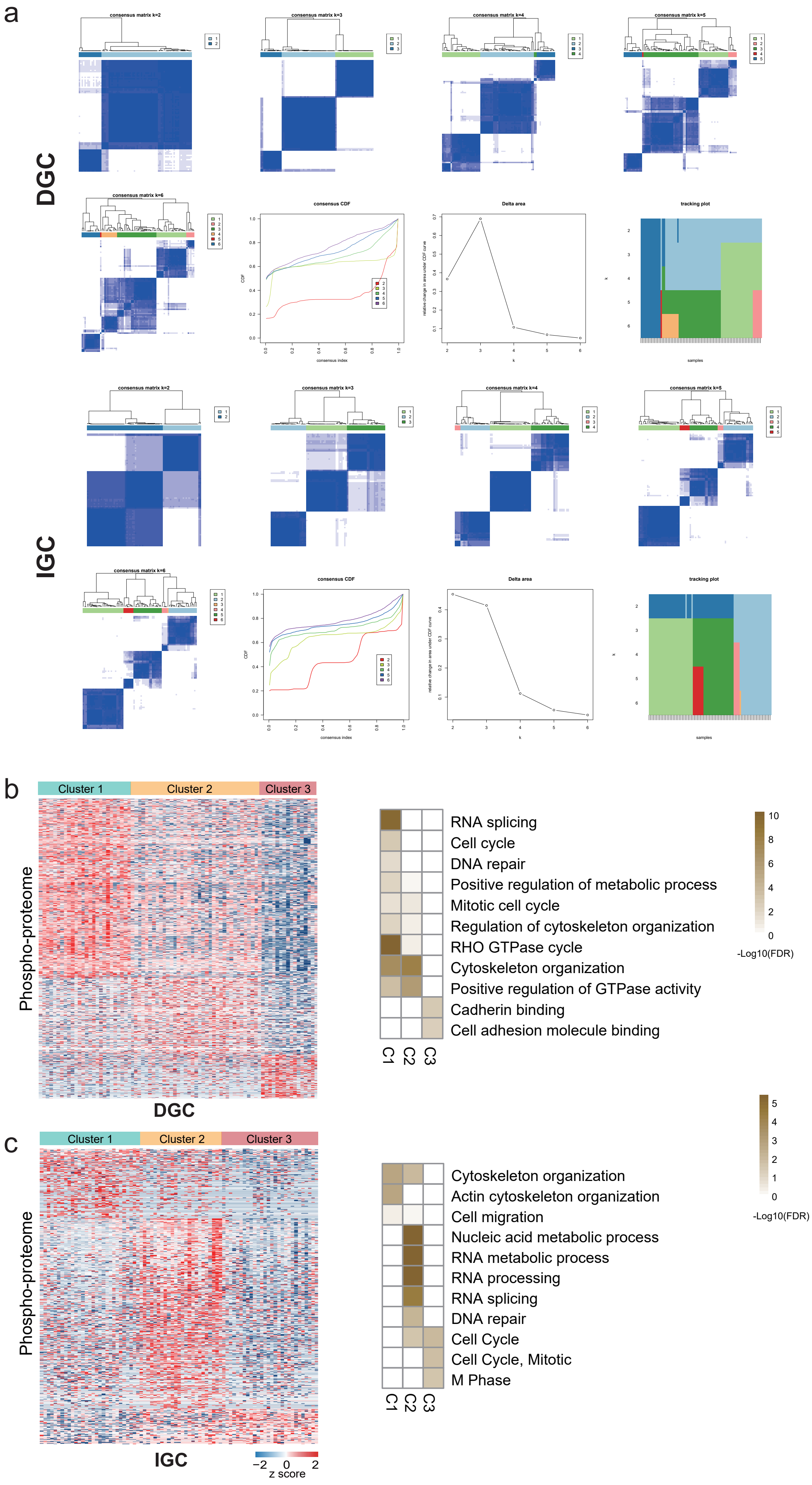

Supplementary Figure 9. **GC subtypes based on phospho-proteomic data**. Related to Figure 6. **a**, Consensus clustering results of phospho-proteomic subtypes of DGC and IGC. **b**, The phospho-sites up-regulated in three subtypes and enriched pathways in three subtypes of DGC. **c**, The phospho-sites up-regulated in three subtypes and enriched pathways in three subtypes of IGC.

Supplementary Figure 10

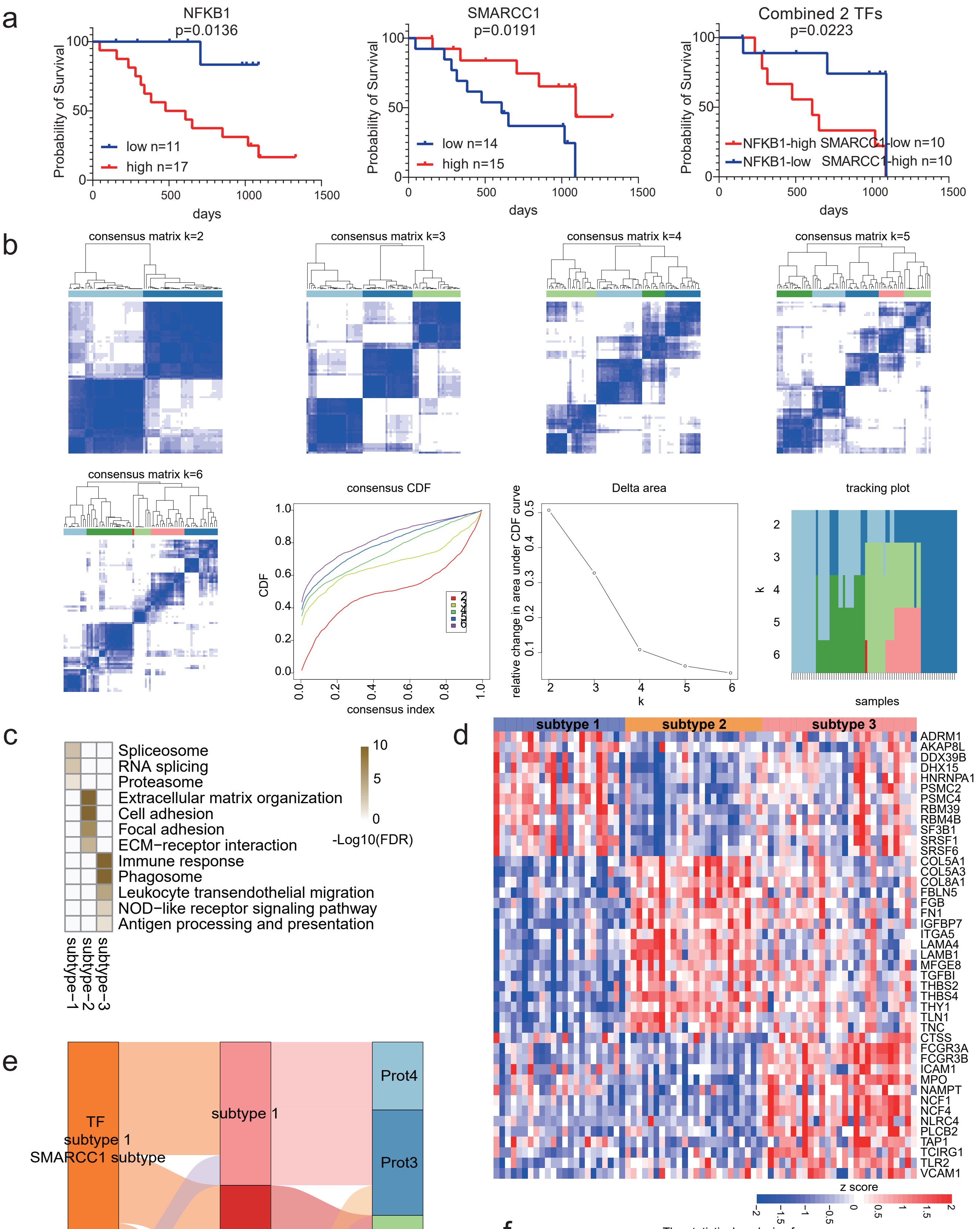

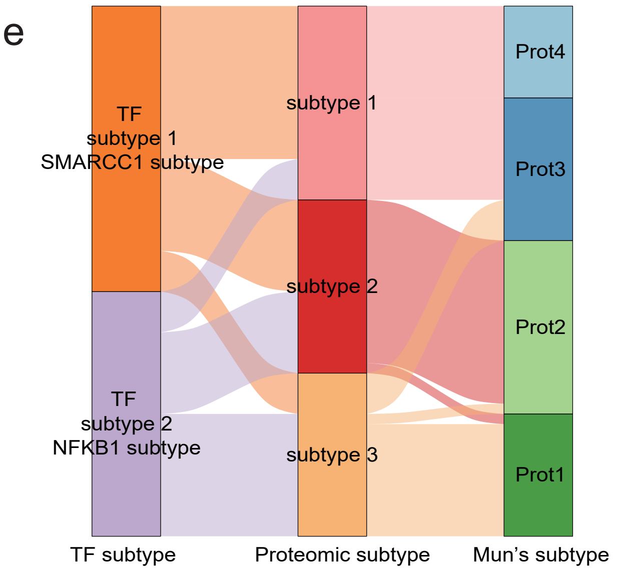

f

The statistical analysis of
the correspondence among validation cohort subtypes

Subtypes	Patient numbers		
Proteomic subtypes	subtype-1	subtype-2	subtype-3
TF subtypes 1	15	9	4
TF subtypes 2	4	8	12
p value			0.006
Proteomic subtypes	subtype-1	subtype-2	subtype-3
Mun's subtype Prot1	0	2	20
Mun's subtype Prot1	1	21	1
Mun's subtype Prot1	11	1	6
Mun's subtype Prot1	11	0	0
p value			1.53E-20

Patient numbers are shown in table. P values by two-sided chi-square test.

Supplementary Figure 10. Validation of GC subtypes in Mun's cohort. Related to Figure 6. **a**, Prognostic analysis about two TFs NFKB1 and SMARCC1. NFKB1: n (low) = 11 and n (high) = 17 biologically independent samples. SMARCC1: n (low) = 14 and n (high) = 15 biologically independent samples. Combined: n (NFKB1 high and SMARCC1 low) = 10 and n (NFKB1 low and SMARCC1 high) = 10 biologically independent samples. The p values were from Log rank test. **b**, Consensus clustering result of Mun's proteomic data. **c**, Pathway enrichment results in three subtypes. **d**, The feature proteins of three subtypes. **e**, Sankey diagram depicting the association of samples classified into proteomic subtypes (center) with TF subtypes (left) and the Mun's subtypes (right). **f**, The statistical analysis of the correspondence among validation cohort subtypes. Patient numbers were shown in table. The p values were from two-sided chi-square test.

Supplementary Figure 11

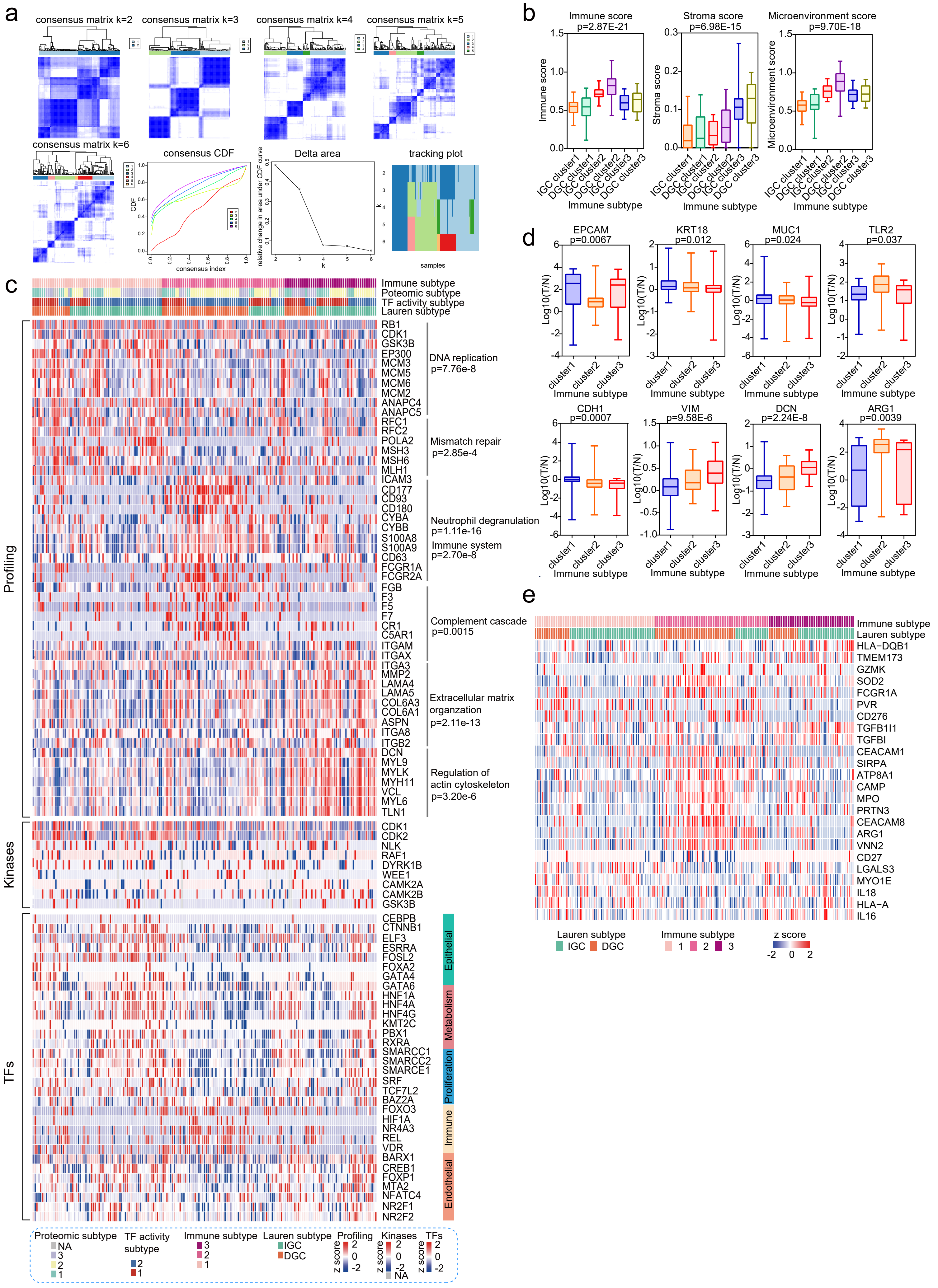

Supplementary Figure 11. **Immune-based subtypes of GC.** Related to Figure 7. **a**, Consensus clustering analysis of xCell scores identified three subtypes in GC. **b**, xCell scores of six immune clusters. P values were from two-sided ANOVA. n (DGC cluster 1) = 20, n (DGC cluster 2) = 46, (DGC cluster 3) = 17, n (IGC cluster 1) = 49, n (IGC cluster 2) = 19, and n (IGC cluster 3) = 32 biologically independent samples. Boxplots showed median (central line), upper and lower quartiles (box limits), min to max range. **c**, Representative differentially expressed proteins, kinases, and TF activities in the featured pathways of immune clusters. The p values were from KEGG pathway enrichment analysis (Fisher's exact test). **d**, Representative lineage-specific protein markers across immune clusters. P values were from two-sided ANOVA. n (cluster 1) = 69, n (cluster 2) = 65, and n (cluster 3) = 49 biologically independent samples. Boxplots showed median (central line), upper and lower quartiles (box limits), min to max range. **e**, Expression of 26 immunotherapeutic targets in clinical development. Source data are provided as a Source Data file.

Supplementary Figure 12

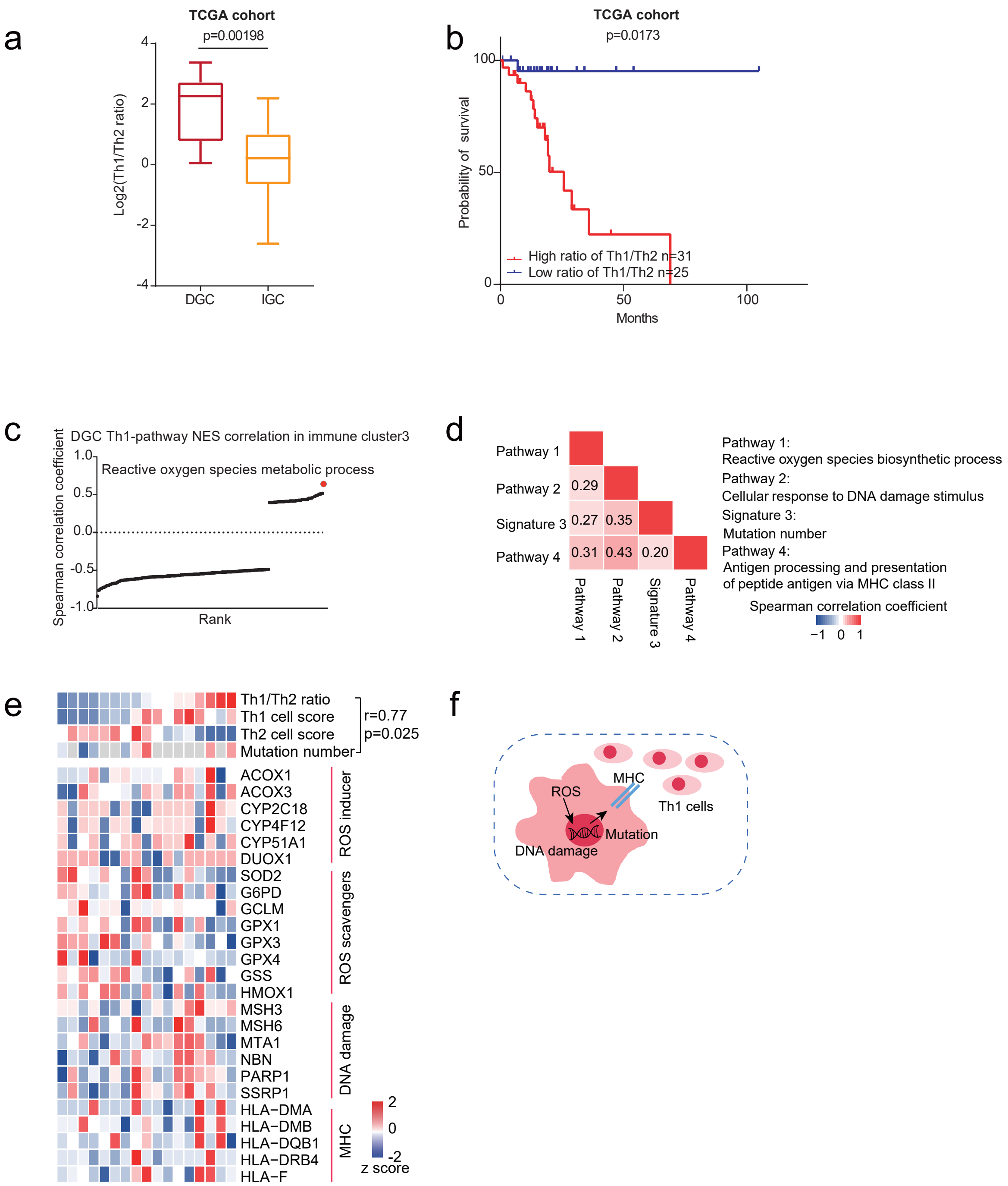

Supplementary Figure 12. **The correlation between Th1/Th2 ratio and ROS.** Related to Figure 7. **a**, Th1/Th2 ratio was higher in DGC than IGC in TCGA cohort. n (DGC) = 9 and n (IGC) = 28 biologically independent samples. Boxplots showed median (central line), upper and lower quartiles (box limits), min to max range. The p value was calculated using two-sided Wilcoxon rank-sum test. **b**, Patients with high Th1/Th2 ratio had poor prognoses in TCGA cohort. n (low) = 25 and n (high) = 31 biologically independent samples. The p values were from Log rank test. **c**, The correlation between Th1 score and GSEA pathway NES in DGC immune cluster 3. **d**, The correlation among pathways or signatures in DGC immune cluster 3. **e**, A list of proteins in related pathways in DGC immune cluster 3. The correlation coefficient and p value were from Spearman's correlation test. **f**, Summary of Th1 cells recruitment mechanism in DGC immune cluster 3. Source data are provided as a Source Data file.
