# Supplementary material for: Multilevel proteomic analyses reveal molecular diversity between diffuse-type and intestinal-type gastric cancer
Source: Nat Commun. 2023 Feb 14;14:835. doi: 10.1038/s41467-023-35797-6 (PMC9929250; doi:10.1038/s41467-023-35797-6)
Supplement: Supplementary file 2 — Description of Additional Supplementary Files [file 41467_2023_35797_MOESM2_ESM.pdf]

Description of Additional Supplementary Files

File Name: Supplementary Data 1
Description: Clinical characteristics of GC patients.

File Name: Supplementary Data 2
Description: The result of targeted sequencing.

File Name: Supplementary Data 3
Description: Lists of differentially expressed proteins in tumor tissues and NATs.

File Name: Supplementary Data 4
Description: Lists of differentially expressed proteins in DGC and IGC.

File Name: Supplementary Data 5
Description: Characteristics of proteomic subtypes.

File Name: Supplementary Data 6
Description: TF activity characteristics of 196 GC patients.

File Name: Supplementary Data 7
Description: Immune infiltration signatures in GC.
