# Supplementary material for: Multilevel proteomic analyses reveal molecular diversity between diffuse-type and intestinal-type gastric cancer
Source: Nat Commun. 2023 Feb 14;14:835. doi: 10.1038/s41467-023-35797-6 (PMC9929250; doi:10.1038/s41467-023-35797-6)
Supplement: Supplementary file 10 — Reporting Summary [file 41467_2023_35797_MOESM10_ESM.pdf]

nature portfolio

Corresponding author(s): Chen Ding

Last updated by author(s): Nov 20, 2022

Reporting Summary

Nature Portfolio wishes to improve the reproducibility of the work that we publish. This form provides structure for consistency and transparency in reporting. For further information on Nature Portfolio policies, see our Editorial Policies and the Editorial Policy Checklist.

Statistics

For all statistical analyses, confirm that the following items are present in the figure legend, table legend, main text, or Methods section.

n/a	Confirmed	
☐	☒	The exact sample size (*n*) for each experimental group/condition, given as a discrete number and unit of measurement
☐	☒	A statement on whether measurements were taken from distinct samples or whether the same sample was measured repeatedly
☐	☒	The statistical test(s) used AND whether they are one- or two-sided<br>*Only common tests should be described solely by name; describe more complex techniques in the Methods section.*
☒	☐	A description of all covariates tested
☐	☒	A description of any assumptions or corrections, such as tests of normality and adjustment for multiple comparisons
☐	☒	A full description of the statistical parameters including central tendency (e.g. means) or other basic estimates (e.g. regression coefficient) AND variation (e.g. standard deviation) or associated estimates of uncertainty (e.g. confidence intervals)
☐	☒	For null hypothesis testing, the test statistic (e.g. *F*, *t*, *r*) with confidence intervals, effect sizes, degrees of freedom and *P* value noted<br>*Give P values as exact values whenever suitable.*
☒	☐	For Bayesian analysis, information on the choice of priors and Markov chain Monte Carlo settings
☒	☐	For hierarchical and complex designs, identification of the appropriate level for tests and full reporting of outcomes
☐	☒	Estimates of effect sizes (e.g. Cohen's *d*, Pearson's *r*), indicating how they were calculated

Our web collection on statistics for biologists contains articles on many of the points above.

Software and code

Policy information about availability of computer code

Data collection	MS raw files were processed with the Firmiana(version 1.0) proteomics workstation. Raw files were searched against the NCBI human Refseq protein database (released on 04-07-2013, 32,015 entries) in Mascot search engine (version 2.3, Matrix Science Inc)
Data analysis	Statistical analyses, included Chi-square test, Fisher's exact test, Wilcoxon rank-sum test, Wilcoxon signed-rank test, One-way anova, Hierarchical clustering analysis, Benjamini-Hochberg (BH) correction, and Principal component analysis (PCA), were realized by R (v4.0.4). Analysis on dominant biological processes was performed with the Gene Set Enrichment Analysis (GSEA) software (v4.1.0) or R package clusterProfiler (v3.18.1). Survival analysis was performed with the R package Survival (v3.2-11) or GraphPad 6.0. Consensus clustering was performed using the R package ConsensusClusterPlus (v1.54.0). Cell cycle phase analysis was performed using the R package Seurat (v4.0.1).For the optimal cutoff point in the K-M analysis of certain protein, we used "survminer" package (v0.4.9). The changes in a kinase's activity were estimated by Kinase-Substrate Enrichment Analysis (KSEA) app(v1.0).

For manuscripts utilizing custom algorithms or software that are central to the research but not yet described in published literature, software must be made available to editors and reviewers. We strongly encourage code deposition in a community repository (e.g. GitHub). See the Nature Portfolio guidelines for submitting code & software for further information.

Data

Policy information about availability of data

All manuscripts must include a data availability statement. This statement should provide the following information, where applicable:

- Accession codes, unique identifiers, or web links for publicly available datasets
- A description of any restrictions on data availability
- For clinical datasets or third party data, please ensure that the statement adheres to our policy

The MS raw data generated in this study have been deposited in the ProteomeXchange Consortium (http://proteomecentral.proteomexchange.org/cgi/GetDataset?ID=PXD038214, dataset identifier: PXD038214) via the iProX partner repository under accession code IPX0004428000 (https://www.iprox.cn/page/project.html?id=IPX0004428000). The normalized proteome, phospho-proteome, and TF activity data matrices are available under this accession. The MS raw data of anti-PD1 group have been deposited in the ProteomeXchange Consortium (http://proteomecentral.proteomexchange.org/cgi/GetDataset?ID=PXD038188, dataset identifier: PXD038188) via the iProX partner repository under accession code IPX0004819000 (https://www.iprox.cn/page/project.html?id=IPX0004819000). The targeted exome sequencing data are available in the GSA (Genome Sequence Archive, https://ngdc.cncb.ac.cn/gsa-human/) under restricted access HRA002466 (https://ngdc.cncb.ac.cn/gsa-human/browse/HRA002466) and HRA003612 (fastq files) (https://ngdc.cncb.ac.cn/gsa-human/browse/HRA003612) for data privacy laws related to patient consent for data sharing, access can be obtained by the Request Data steps in GSA database website or contacting corresponding author. The approximate response time for accession requests is about 2 weeks. Once access has been granted, the data will be available to download for 3 months. The TCGA publicly available data used in this study are available in the Genomic Data Commons Data Portal under accession code TCGA-STAD (https://portal.gdc.cancer.gov/). The remaining data are available within the Article, Supplementary Information or Source Data file. Source data are provided with this paper.

Human research participants

Policy information about studies involving human research participants and Sex and Gender in Research.

Reporting on sex and gender	This study included 52 females and 144 males. The detailed information was provided in Supplemental table 1.
Population characteristics	This research inncluded 83 cases of diffuse-type gastric cancer (DGC), 102 cases of intestinal-type gastric cancer (IGC) and 11 cases of mixed gastric cancer (MGC). The detailed information was provided in Supplemental table 1.
Recruitment	The samples were obtained from Peking University Cancer Hospital, Xijing Hospital, and Chinese PLA General Hospital. These 196 patients underwent total or subtotal gastrectomy between 2012 and 2015, and no patient in this cohort was treated with neoadjuvant chemotherapy or chemo-radiation therapy before operation. There were 144 patients (~75%) received chemotherapy after surgery. Whether patients receive chemotherapy or not was based on the clinical guidelines, patients' prognosis and the patients' willingness. With or without chemotherapy in this research was defined as with or without at least one cycle of adjuvant chemotherapy. Demographics, histopathologic information, primary tumor location, treatment details including chemotherapy drugs, doses and routes of administration, and outcome parameters were collected and presented in Supplemental table 1.
Ethics oversight	Ethical approval was obtained from Peking University Cancer Hospital Medical Research Ethics Committee, Xijing Hospital Medical Research Ethics Committee, and Chinese PLA General Hospital Medical Research Ethics Committee.

Note that full information on the approval of the study protocol must also be provided in the manuscript.

Field-specific reporting

Please select the one below that is the best fit for your research. If you are not sure, read the appropriate sections before making your selection.

[x] Life sciences [ ] Behavioural & social sciences [ ] Ecological, evolutionary & environmental sciences

For a reference copy of the document with all sections, see nature.com/documents/nr-reporting-summary-flat.pdf

Life sciences study design

All studies must disclose on these points even when the disclosure is negative.

Sample size	No statistical method was used to pre-determine the sample size. The sample size was based on published papers in the proteomic field (PMID: 29520031; 30814741; 32649877). Saturation curve (Supplemental Figure 1 F) and the statistical differences in the subtypes (Figure 4) provide the rationale for sufficiency of the sample sizes.
Data exclusions	For samples data, the distribution of median values was used to discriminate the samples with insufficient protein or phospho-site detected. The samples with median values which were larger than upper quartile + 1.5 IQR (interquartile range) would be excluded from further analyses. To evaluate the comparability of data, we compared the data distribution with boxplots and density curves. Samples with a clear bimodal distribution of protein quantification would be excluded from further analyses. Furthermore, QC results required both of tumor tissues and paired NATs passed QC procedures.
Replication	The replicated analyses of QC(quality control) standards (tryptic digestions of the 293T cell lysate) were perform to evaluate the mass

	spectrometer platforms. The high average spearman's correlation coefficient showed the stability of our MS platforms. Besides, the proteomic subtypes of DGC and IGC were identified by consensus clustering in bioinformatic analysis, which repeated clustering 1,000 times. These provided the stable classification. For intergroup comparison, there were repeats in each group, which avoided the heterogeneity among patients. The patient number in each group was indicated in Results and figure legends.
Randomization	We performed consensus clustering for the proteomic subtyping based on R package ConsensusClusterPlus (v1.54.0). Samples were clustered with 1,000 resampling repetitions in the range of 2 to 6 clusters. The consensus CDF and delta plots provided the clearest separation among the clusters. Then, samples were allocated into different groups. All the survival analyses of proteomic subtyping were adjusted by other clinical covariates including gender, age, TNM stage and chemotherapy.
Blinding	The investigators who performed sample processing and measured protein expression by mass spectrometry were blinded to patient information.

Reporting for specific materials, systems and methods

We require information from authors about some types of materials, experimental systems and methods used in many studies. Here, indicate whether each material, system or method listed is relevant to your study. If you are not sure if a list item applies to your research, read the appropriate section before selecting a response.

Materials & experimental systems

n/a	Involved in the study	
☒	☐	Antibodies
☐	☒	Eukaryotic cell lines
☒	☐	Palaeontology and archaeology
☒	☐	Animals and other organisms
☒	☐	Clinical data
☒	☐	Dual use research of concern

Methods

n/a	Involved in the study	
☒	☐	ChIP-seq
☒	☐	Flow cytometry
☒	☐	MRI-based neuroimaging

Eukaryotic cell lines

Policy information about cell lines and Sex and Gender in Research

Cell line source(s)	Human HEK293T (Cat# CRL-11268 from ATCC; RRID: CVCL_QW54) were obtained.
Authentication	Cells validation using short tandem repeat markers (STR) were performed by Meixuan Biological Science and Technology Ltd. (Shanghai). In detail, these cell lines were firstly tested cell species by PCR method using extracted total genomic DNA, and examined by STR profiling. Then, STR data were analyzed using the DSMZ (German Collection of Microorganisms and Cell Cultures) online STR database (http://www.dsmz.de/fp/cgi-bin/str.html).
Mycoplasma contamination	Cell line was tested negative for mycoplasma contamination.
Commonly misidentified lines (See ICLAC register)	No commonly misidentified cell lines were used.
